# Degradomic Identification of Membrane Type 1-Matrix Metalloproteinase as an ADAMTS9 and ADAMTS20 Substrate

**DOI:** 10.1016/j.mcpro.2023.100566

**Published:** 2023-05-09

**Authors:** Sumeda Nandadasa, Daniel Martin, Gauravi Deshpande, Karyn L. Robert, M. Sharon Stack, Yoshifumi Itoh, Suneel S. Apte

**Affiliations:** 1 Department of Biomedical Engineering, Cleveland Clinic Lerner Research Institute, Cleveland, Ohio, USA; 2 Department of Pediatrics, University of Massachusetts Medical School, Worcester, Massachusetts, USA; 3 Imaging Core Facility, Cleveland Clinic Lerner Research Institute, Cleveland, Ohio, USA; 4 Department of Chemistry and Biochemistry and Harper Cancer Center, University of Notre Dame, Notre Dame, Indiana, USA; 5 Kennedy Institute for Rheumatology, University of Oxford, Oxford, UK

**Keywords:** ADAM, ADAMTS, cleavage, degradome, degradomics, focal adhesion, gene-editing-N-terminomics, metalloprotease, MMP, MT1-MMP, protease, proteolysis, substrate

## Abstract

The secreted metalloproteases ADAMTS9 and ADAMTS20 are implicated in extracellular matrix proteolysis and primary cilium biogenesis. Here, we show that clonal gene-edited RPE-1 cells in which *ADAMTS9* was inactivated, and which constitutively lack *ADAMTS20* expression, have morphologic characteristics distinct from parental RPE-1 cells. To investigate underlying proteolytic mechanisms, a quantitative terminomics method, terminal amine isotopic labeling of substrates was used to compare the parental and gene-edited RPE-1 cells and their medium to identify ADAMTS9 substrates. Among differentially abundant neo-amino (N) terminal peptides arising from secreted and transmembrane proteins, a peptide with lower abundance in the medium of gene-edited cells suggested cleavage at the Tyr^314^-Gly^315^ bond in the ectodomain of the transmembrane metalloprotease membrane type 1-matrix metalloproteinase (MT1-MMP), whose mRNA was also reduced in gene-edited cells. This cleavage, occurring in the MT1-MMP hinge, that is, between the catalytic and hemopexin domains, was orthogonally validated both by lack of an MT1-MMP catalytic domain fragment in the medium of gene-edited cells and restoration of its release from the cell surface by reexpression of ADAMTS9 and ADAMTS20 and was dependent on hinge *O*-glycosylation. A C-terminally semitryptic MT1-MMP peptide with greater abundance in WT RPE-1 medium identified a second ADAMTS9 cleavage site in the MT1-MMP hemopexin domain. Consistent with greater retention of MT1-MMP on the surface of gene-edited cells, pro-MMP2 activation, which requires cell surface MT1-MMP, was increased. MT1-MMP knockdown in gene-edited ADAMTS9/20-deficient cells restored focal adhesions but not ciliogenesis. The findings expand the web of interacting proteases at the cell surface, suggest a role for ADAMTS9 and ADAMTS20 in regulating cell surface activity of MT1-MMP, and indicate that MT1-MMP shedding does not underlie their observed requirement in ciliogenesis.

## Highlights

• ADAMTS9-deficient RPE-1 cells have impaired attachment to a glass substrate.
• ADAMTS9 and ADAMTS20 release the MT1-MMP catalytic domain from the cell surface.
• Increased cell surface MT1-MMP increases pro-MMP2 activation and collagenolysis.
• MT1-MMP knockdown restores attachment of ADAMTS9-deficient RPE-1 cells.

## Graphical Abstract

**Figure:**
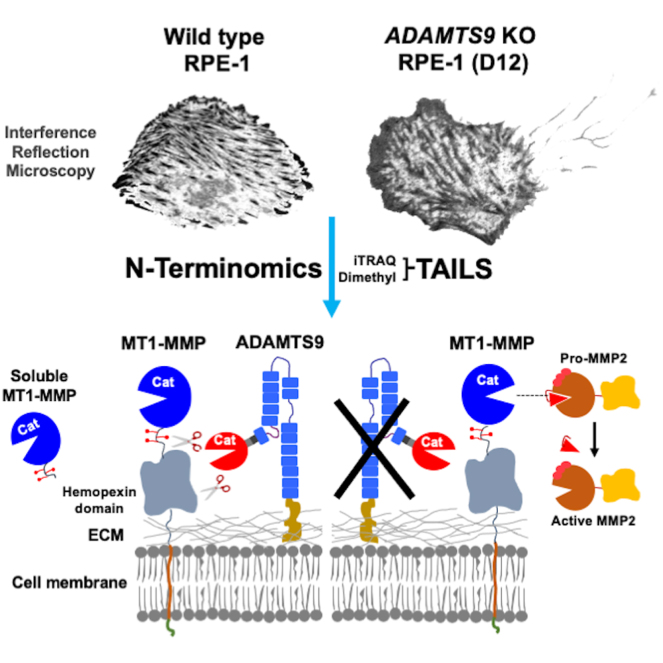

## In Brief

ADAMTS9 and ADAMTS20 are homologous secreted proteases implicated in extracellular matrix proteolysis and ciliogenesis, but few relevant substrates of these proteases are known. Quantitative terminomics comparison of RPE-1 cells with inactivation of *ADAMTS9* and parental RPE-1 cells identified transmembrane protease MT1-MMP (MMP14) as a novel ADAMTS9 substrate. Enhanced cell surface MT1-MMP activity in *ADAMTS9* gene–edited cells contributes to their adhesion defect but not lack of cilia. One physiological function of ADAMTS9/20 may be to dampen cell surface MT1-MMP activity.

Secreted and cell surface proteases undertake proteolytic modification of pericellular extracellular matrix (ECM), which is relevant to cell adhesion and migration, as well as shedding of cell surface and transmembrane proteins, whose effects include activation or inactivation of adhesion molecules, membrane-bound ligands, receptors, and proteases (1). Two classes of zinc metalloproteases (metzincins) are prominent in cell surface proteolysis, that is, a disintegrin-like and metalloproteinase domain(ADAM) proteases and matrix metalloproteinases (MMPs). ADAMs are single-pass type I membrane-spanning proteins, as are four membrane-type MMPs (MT-MMPs), whereas two other cell surface MMPs (MT4-MMP and MT6-MMP) are inserted into the cell membrane *via* glycosylphosphatidylinositol anchors. Membrane localization of these proteases is a major determinant of their activity at the cell surface. A disintegrin-like and metalloproteinase domain with thrombospondin type 1 repeats (ADAMTS) proteases comprise a distinct class of secreted metalloproteases (2). Most known ADAMTS substrates are ECM components (2). ADAMTS proteases have an affinity for the cell surface and/or pericellular matrix and like ADAMs and MT-MMPs, also act in a cell proximate manner. ADAMTS1 and ADAMTS6, which bind heparan-sulfate proteoglycans (3), were shown to cleave the syndecan-4 ectodomain (4, 5), but transmembrane proteins are generally not thought to be preferred ADAMTS substrates.

ADAMTS9 and its mammalian homolog ADAMTS20 are evolutionarily conserved proteases with readily recognizable orthologs in *Caenorhabditis elegans* and *Drosophila melanogaster* (6, 7). These large proteases (predicted molecular mass ∼215,000–225,000 depending on glycosylation) are distinguished both by having more thrombospondin type 1 repeats than other ADAMTS proteases and by a unique C-terminal domain (the Gon-1 domain) (7). In mice, *Adamts9* is expressed from early embryo development onward (7, 8, 9, 10, 11, 12). In adult tissues, it is a product of capillary and venous endothelium and is expressed by several other cell types (9, 13). Analysis of mouse *Adamts9* null embryos, mouse embryos homozygous for a hypomorphic *Adamts9* gene-trap allele, and of conditional mouse *Adamts9* mutants demonstrated essential roles for ADAMTS9 in gastrulation, cardiovascular, eye, and umbilical cord development and together with ADAMTS20, in neural tube and palate closure, interdigital web regression, and skin pigmentation (9, 10, 11, 12, 14, 15, 16, 17, 18). In worms, ADAMTS9 is implicated in gonad development (19, 20), in flies in tracheal, salivary gland, and germ cell development (21) and in zebrafish, early germ cell migration and ovarian development (22, 23). ADAMTS9 and ADAMTS20 are necessary for formation of the primary cilium (11), a cellular organelle of postmitotic cells that transmits signaling by hedgehog proteins and other morphogens, and recessive *ADAMTS9* mutations were found in the human ciliopathies nephronophthisis and Joubert syndrome (24, 25). Some morphogenetic defects of *Adamts9* and *Adamts20* mouse mutants may reflect their cooperation (with each other and other ADAMTS proteases) in processing the chondroitin-sulfate proteoglycan versican, an important ECM component of early- to mid-gestation embryos (reviewed in (24, 25)), which accumulates in some tissues of *Adamts9/20* mutant embryos (11, 12). Recessive *Adamts20* mutations in mice (*Belted* [*Bt*] mutant) lead to a depigmented belt in the torso (26, 27) and to partially penetrant soft-tissue syndactyly and cleft palate (10, 12, 15, 17), which also occur in dogs with an *ADAMTS20* variant (28). Mice with limb-specific conditional deletion of ADAMTS9 develop soft-tissue syndactyly (15). This defect is also present in mice with elimination of the canonical ADAMTS cleavage site in versican, validating versican as a key ADAMTS9 and ADAMTS20 substrate in digit separation (29). However, mice with cleavage-resistant versican lack other defects observed in *Adamts9* and *Adamts20* mouse mutants suggesting that there are additional substrates yet to be defined. Aggrecan and fibronectin, both components of the ECM, are confirmed ADAMTS9 substrates (29). Degradomic analysis of skin from a hypomorphic mouse *Adamts9* mutant identified several ECM proteins as putative ADAMTS9 substrates but these remain unvalidated (30).

Cooperative functions of ADAMTS9 and ADAMTS20 elicited in the combined mouse mutants suggested that their proteolytic activities may overlap. Furthermore, impaired ciliogenesis in gene-edited retinal pigment epithelium-1 (RPE-1) cells lacking ADAMTS9 was restored by ADAMTS20 transfection (11). Identification of ADAMTS9 and ADAMTS20 substrates, in the ECM, at the cell surface or in ciliogenesis pathways is therefore crucial to define the underlying molecular mechanisms of these important secreted proteases. Here, we used proteomics to investigate changes in the cell–substratum interface of an ADAMTS9-deficient clonal RPE-1 line (designated as clone D12), which was generated by CRISPR-Cas9 editing of RPE-1 cells (11). To explain the observed morphologic differences, we applied the terminomics strategy terminal amine isotopic labeling of substrates (TAILS) (31) and compared the degradomes of these cells with parental RPE-1 cells. RPE-1 cells and D12 cells constitutively lack ADAMTS20 expression (11), maximizing the potential for discovery of ADAMTS9/20 substrates. Moreover, D12 cells have an unequivocal ADAMTS9/20-dependent phenotype, that is, impaired formation of the primary cilium. The findings of the study demonstrate a role for ADAMTS9 in regulating the activity of MT1-MMP, an important cell surface metalloprotease involved in cell migration, tumor cell invasion, and numerous other processes (32), including potentially cilium length regulation.

## Experimental Procedures

### Cell Culture, Recombinant DNA, and siRNA Transfections

WT and *ADAMTS9* KO (D12) RPE-1 cells (ATCC, CRL-4000) were cultured in Dulbecco's modified Eagle's medium (DMEM) F/12 culture medium containing 10% fetal bovine serum, 50 U/ml penicillin/streptomycin, and 200 μg/ml hygromycin B (Millipore Sigma, Cat. # 400052) as previously described (11). HEK293T cells (ATCC, CRL-1573) were cultured in DMEM with 10% fetal bovine serum and 50 U/ml penicillin/streptomycin. Plasmids encoding full-length human ADAMTS9 and mouse ADAMTS20 and their catalytically inactive mutants (E/A) in pcDNA3.1 Myc/His vector were previously described (11). WT or catalytically inactive human MT1-MMP constructs with an N-terminal Flag tag between the propeptide and catalytic domain were previously described (33). Human MT1-MMP hinge *N-* and *O*-glycosylation mutants T299A (pCR-CHO-3/f), T299A/T300A/S301A (pCR-CHO-3/f), T291A/N311A (pCR-NACHO-1/f), T299A/T300A/S301A/N311A (pCR-NACHO-3/f), and T291A/T299A/T300A/S301A (pCR-NACHO-4/f) were previously described (34). DNA transfections were carried out using PEI Max transfection reagent. siRNA targeting human MT1-MMP (Ambion, catalog no. AM51331) or a control siRNA (Invitrogen, catalog no. 4390843) was transfected into WT or D12 RPE-1 cells cultured in 8-chamber slides as previously described (11) using Lipofectamine RNAiMAX reagent (Thermo Fisher Scientific Cat# 13778100).

### Western Blotting and Gelatin Zymography

One millileter of serum-free, conditioned medium from RPE-1 or HEK293T cells cultured in 6-well plates was collected 48 h after transfection, and the cell layers were harvested using 300 μl PBS containing 1% Triton X-100 and protease inhibitor complex. 6X Laemmli sample buffer containing β-mercaptoethanol was used to reduce 50 μl of each sample for Western blot analysis after 10% SDS PAGE. For Western blots, anti-FLAG polyclonal antibody (Sigma, 1:1000 dilution) and anti-human MT1-MMP rabbit mAb EP1264Y against the catalytic domain (Abcam, catalog no. ab51074, 1:1000 dilution) were used followed by anti-rabbit secondary antibodies (Li-COR) at 1:15,000 dilution, and the membranes were imaged using a Li-COR Odyssey CLx or a Li-COR Odyssey M infrared imager (Li-COR).

For gelatin zymography, 1 ml of serum-free medium from parental RPE-1 or D12 cells (n = 4 cultures for both) was collected after 48 h culture in a 12-well plate, and EDTA-free protease inhibitor (Roche, catalog no. 11836170001) was added. Medium was concentrated to ∼200 μl using a 10 kDa molecular weight cut-off spin column (Amicon, catalog no. UFC501096), and 0.3 μg of total protein was loaded in each well using 5X Laemmli sample buffer without β-mercaptoethanol. Gelatin zymography was carried out using 10% SDS-polyacrylamide gels containing 1% gelatin (Novex, Thermo Fisher Scientific, catalog no. ZY00102BOX), following the manufacturer’s protocol. Following a 10 min wash with water, the gels were stained for 1 h with GelCode Blue (Thermo Fisher Scientific, catalog no. 24590) and destained for at least 2 h with distilled water. Digestion bands were imaged with the LiCOR Odyssey Clx imager and quantified using ImageJ densitometry software (NIH, ImageJ.org).

### TAILS Sample Preparation and Mass Spectrometry

Proteins from lysates and 5 ml of phenol red-free–, serum-free–conditioned medium of confluent parental RPE-1 and D12 cells were analyzed by TAILS using N-terminal labeling with iTRAQ or reductive dimethylation, respectively. For making lysates, cells were scraped from 10 cm dishes at confluence, washed with PBS, and pelleted by centrifugation for 5 min. Cells were lysed by addition of 2.35 ml of 0.05% SDS, 10 mM DTT in 100 mM Hepes, and 10 mM EDTA, pH 8 with protease inhibitors (cOmplete mini, Millipore Sigma) to the pellet, followed by tip probe sonication for 80 s at a 20% amplitude using a Q-500 sonicator (Qsonica). DNA and RNA were degraded using 2 μl of benzonase (EMD Millipore Corp, catalog no. 70746-10KUN) at room temperature for 20 min, and acetone-methanol precipitation was performed to precipitate proteins. This pellet was resuspended in 2.5 M GuHCl and 250 mM Hepes. Protein quantity was determined using a 592 nm bicinchoninic acid assay (Thermo Fisher Scientific, catalog no. 23225) and normalized to 250 μg per channel. Samples were reduced and alkylated using 10 mM tris(2-carboxyethyl) phosphine and 25 mM iodoacetamide (IAA), respectively. Each sample was labeled with two units of each tag by adding one sample volume of dimethyl sulfoxide to each iTRAQ label (Sciex, catalog no.4390812) before adding the total solution volume to each sample. Four iTRAQ channels were used to label parental RPE-1 cell lysates and four to label D12 lysates for 2 h in the dark at room temperature before quenching with ammonium bicarbonate to a final concentration of 100 mM. The iTRAQ-labeled samples were combined, and proteins were isolated by acetone-methanol precipitation.

The medium was passed through a 10-μm filter to remove cell debris and concentrated to 10-fold using a 3 kDa molecular weight cut-off spin filter (Amicon, catalog no. UFC500396). Protein was quantified as above and normalized to 500 μg per sample. Each sample was reduced and alkylated using 5 mM DTT and 20 mM IAA, respectively, and excess IAA was quenched using an additional 15 mM DTT. Samples were labeled with 40 mM isotopically distinct dimethyl formaldehyde (light, CH_2_O, for the parental RPE-1 cells (Cambridge Isotope Laboratories, Inc, catalog no.ULM-9498-100) and heavy, (^18^CD_2_O) for D12 (Sigma-Aldrich, catalog no. 596388-1G)), added simultaneously with 20 mM NaBH_3_CN, and incubated overnight at 37 °C. Fresh formaldehyde and NaBH_3_CN were added the following day for 1 h. The reaction was quenched in a final concentration of 100 mM Tris–HCl at 37 °C for 2 h. Paired heavy- and light-labeled replicate samples were then mixed, and the proteins were isolated by chloroform-methanol precipitation.

Precipitated pellets were solubilized in 100 mM NaOH and 500 mM Hepes pH 7.5. Trypsin was added at a 1:100 (protease: protein) ratio and incubated at 37 °C overnight. A 30 μg aliquot was removed from each sample (pre-TAILS sample), and the remaining protein was mixed with hyperbranched polyglycerol-aldehydes (Flintbox, https://www.flintbox.com/public/project/1948/) at a 5:1 polymer:protein ratio overnight at 37 °C in the presence of 10 mM NaBH_3_CN. Hyperbranched polyglycerol-aldehyde binds unblocked (*i.e.,* trypsin-generated) amino acid termini, excluding them from the sample and enriches for peptides with blocked/labeled N termini (TAILS peptides). The samples were filtered through a 10 kDa cut-off column (EMD Millipore), and the eluate and pre-TAILS fractions were desalted on a C18 Sep-Pak column (Waters, catalog no. WAT054955) and eluted in 60:40 acetonitrile: 1% TFA. Samples were vacuum-centrifuged until dry and resuspended in 1% acetic acid for mass spectrometry (MS).

The pre-TAILS and TAILS peptides were analyzed on a Thermo Fisher Scientific Fusion Lumos tribrid mass spectrometer system interfaced with a Thermo Ultimate 3000 nano-UHPLC. The HPLC column was a Dionex 15 cm × 75 μm id Acclaim Pepmap C18, 2 μm, 100 Å reversed-phase capillary chromatography column. Five microliters volume of the trypsin-digested extract was injected, and peptides were eluted from the column by an acetonitrile/0.1% formic acid gradient at a flow rate of 0.3 μl/min and introduced in-line into the mass spectrometer over a 120-min gradient. The nanospray ion source was operated at 1.9 kV. The digest was analyzed using a data-dependent method with 35% collision-induced dissociation fragmentation (for the dimethyl-labeled samples) or high-energy collisional dissociation set to 38 (for the iTRAQ-labeled) of the most abundant peptides every 3 s and an isolation window of 0.7 *m/z* for ion-trap tandem MS (MS/MS). Scans were conducted at a maximum resolution of 120,000 for full MS. Dynamic exclusion was enabled with a repeat count of 1, and ions within 10 ppm of the fragmented mass were excluded for 60 s.

### Proteomics Data Analysis

Spectra were searched against the reviewed human database (November 2018, 42,357 entries) using Proteome Discoverer 2.2 (Thermo Fisher Scientific). Peptides were identified using a precursor mass tolerance of 10 ppm and fragment mass tolerance of 0.6 Da with the static modification being carbamidomethyl (C), whereas dynamic modifications included either the light (28.03 Da) or heavy (32.06) dimethyl formaldehyde (N-terminal, K) or iTRAQ 8-plex (N-terminal, K) as well as oxidation (M), deamidation (N), acetylation (N-terminal), and Gln to pyro-Glu N-terminal cyclization. Peptides were validated using a false discovery rate of 1% against a decoy database. Only high confidence proteins (containing peptides at a 99% confidence level or higher) were recorded from each sample for data analysis. N-terminal peptides were identified by the presence of an N-terminal dimethyl label or other blocking entity. Statistical analysis is described in the Experimental Design and Statistical Rationale section.

### Interference Reflection Microcopy

Parental RPE1 or D12 cells, with or without siRNA treatment, were seeded onto glass-bottom dishes (MatTek, catalog no. P35G-0-14-C) in phenol-red–free medium and imaged 30 min or 24 h after seeding. Live imaging was carried out for 2 h at 1 min intervals in a climate-controlled environmental chamber, using a Leica SP8 confocal microscope (Leica Microsystems). Images were acquired using a 40X/1.25 oil immersion objective at zoom of 1.275, 488 nm excitation with a photomultiplier tube (PMT) detector collecting window of 460 nm to 520 nm. PMT gain parameters were set using human dermal fibroblasts cultured on coverslip bottom dishes for 24 h. These parameters were kept constant for imaging WT and siRNA knockdown cells. Two hundred fifty-six gray values are based on the PMT detector (13). Histograms from each cell were used to quantify focal adhesions with the lowest brightness levels (intensity <40) representative of strong focal adhesions (distance between the cell and the plate <2.5 Å).

### Imaging of Substratum Collagen Proteolysis

Eight-well chamber slides were coated with 25 μg/ml DQ collagen type I (Invitrogen, catalog no. D12060) containing a 1:11 ratio of DQ-conjugated to nonconjugated bovine collagen I for 2 h at room temperature and seeded with parental RPE-1 or D12 cells followed by culture for 24 h in serum-free DMEM+F12 culture medium. A coated culture well without seeded cells was used as a control to define the basal (quenched) fluorescence signal of DQ collagen type I. Twenty-four hours after seeding, culture medium was aspirated, and the cell layer was washed once with PBS and fixed with 4% paraformaldehyde (PFA) in PBS containing 0.1% Tween-20 (PBST) for 10 min with agitation. The fixed cell layer was washed three times using PBST and mounted overnight using ProLong Gold mounting medium containing 4',6-diamidino-2-phenylindole, dihydrochloride (DAPI) (Invitrogen, P36931). Unquenched fluorescein DQ-collagen signal was imaged using a Zeiss Axioplan microscope equipped with a Leica DM6200 camera.

### Imaging of Cell Surface MT1-MMP and Quantitative Assessment of Cilia

Parental RPE-1 and D12 cells were seeded in 8-well chamber glass slides for 24 h and were treated with control siRNA or *MMP14* siRNA using Lipofectamine RNAiMAX transfection reagent overnight. Culture medium was removed, and the cell layer was washed with sterile PBS, and the cells were cultured in serum-free DMEM-F12 culture medium for induction of cilia. For MT1-MMP overexpression experiments, 8-chamber glass slides were coated with 5 μg/ml vitronectin (R&D systems, 2308-VN) for 2 h at room temperature prior to being seeded with parental RPE-1 cells. After 24 h culture, the cells were transfected with 100 ng/well of pcDNA3.1 Myc/His empty vector or MT1-MMP plasmid DNA using PEI max transfection reagent (Polysciences, catalog no. 24765). After 6 h treatment, cells were washed with PBS and cultured in serum-free DMEM-F12 medium for 24 h. In both knockdown and overexpression experiments, the cells were harvested after 24 h by washing once with PBS followed by fixation in 4% PFA in PBST for 15 min. Primary cilia were visualized by staining with anti-acetylated alpha tubulin (1:400, Invitrogen, catalog no. 32-2700) and imaged using a Zeiss Axioplan microscope equipped with a Leica DMC6200 camera. Cilium length and percentage was quantified using NIH ImageJ Fiji as previously described (11). MT1-MMP immunostaining was conducted on cells grown in 8-chamber slides and fixed with 4% PFA in PBS (without 0.l% Tween-20), utilizing a 1:400 dilution of the rabbit monoclonal MT1-MMP antibody (EP1264Y) against the catalytic domain (Abcam, catalog no. ab51074). Blocking, primary and secondary antibody staining, and washing steps were conducted under detergent-free conditions. Prior to coverslipping and mounting with the ProLong Gold mounting medium containing DAPI (Invitrogen, P36931), the cells were washed with PBST for 15 min to facilitate permeability for nuclear staining with DAPI.

### Quantitative Reverse Transcriptase PCR

Real time quantitative PCR (RT-qPCR) was performed as described (35). In brief, 2 μg of total RNA harvested from parental RPE-1 and D12 cells utilizing TRIzol reagent (Thermo Fisher Scientific, catalog no. 15596026) was used for complementary DNA synthesis (Applied Biosystems, Thermo Fisher Scientific, catalog no. 4368814). *GAPDH* expression was used for normalizing relative gene expression calculated by the ΔΔCt quantification method using a CFX96 touch real-time PCR detection system (Bio-Rad Laboratories). An unpaired, two-tailed Student *t* test was used to calculate statistical significance. The following primer pairs were used for RT-qPCR analysis. *GAPDH*-F 5′-AGCCTCAAGATCATCAGCAATG-3′, *GAPDH*-R 5′-CTTCCACGATACCAAAGTTGTCAT-3′; *MMP14*-F 5′-CCTTGGACTGTCAGGAATGAGG-3′, *MMP14*-R 5′-TTCTCCGTGTCCATCCACTGGT-3′; *MMP2*-F 5′-AGCGAGTGGATGCCGCCTTTAA-3′, *MMP2*-R 5′-CATTCCAGGCATCTGCGATGAG-3′.

### Experimental Design and Statistical Rationale

Since the experiments compared parental RPE-1 cells and D12 cells only, no additional biological replicates were possible, but technical replicates were used. For cell imaging, three independent wells were used for each cell type and at least ten independent fields were scanned for each. For Western blots and RT-qPCR, n = 3 was used, and for gelatin zymography, n = 4 was used. Western blots and the gelatin zymogram show all the run samples. For TAILS and pre-TAILS experiments, duplex dimethyl labeling was done using pairs of RPE-1 and D12 cells (individual cell cultures). The duplex dimethyl-labeled samples were injected separately for a total of six injections (3 for TAILS and 3 for pre-TAILS) each for the medium and the cell lysate. Each raw file was searched individually using Proteome Discoverer 2.2 software, and the respective experimental datasets were combined for analysis. This number proved to be sufficient for statistical comparison and evaluation of reproducibility and provided statistically significant differences. An unpaired, two-tailed Student *t* test was used to calculate statistical significance of RT-qPCR and Western blot data.

For MS data analysis, peptide groups files were then further analyzed using Perseus 1.6.15.0 (https://maxquant.net/perseus/). The software was used to initially log2 transform normalized peptide abundances. Then, peptides were filtered out based on their presence in at least 2 of 3 technical replicates in at least one group. Missing values were then imputed based on a 1.8 downshift and a width of 0.3, and each dataset was determined visually to maintain a normal distribution based on histogram changes following imputations. Next abundances between groups were compared using a Welch’s *t* test with a Benjamini–Hochberg correction using a *p* value of <0.05 for significance. Peptide annotation was performed using TopFIND (TopFINDER tool, http://clipserve.clip.ubc.ca/topfind/topfinder) to distinguish between neo-N terminal and natural peptides (36, 37, 38). Data visualization utilized GraphPad Prism 9 (www.graphpad.com), PowerPoint, Excel (Microsoft, 2013), and peptide positions were mapped on proteins based on their structure using uniprot.org annotations.

## Results

### Perturbation of Substratum Contacts in ADAMTS9-Deficient RPE-1 cells

**Fig. 1:**
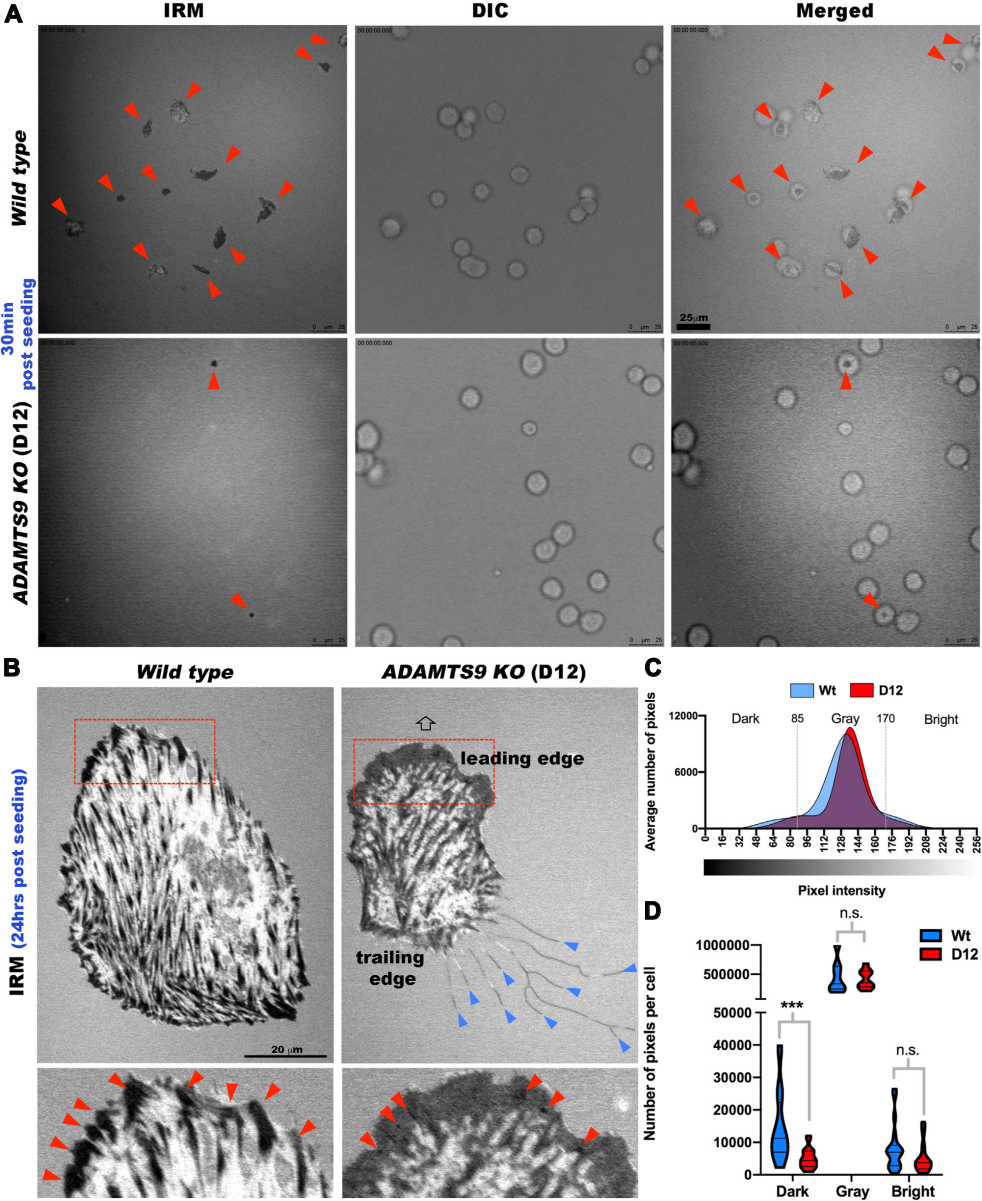
**Altered cell–substratum interface in gene-edited RPE-1 cells lacking ADAMTS9.***A*, still photomicroscopy of concurrent, aligned interference reflection microscopy (IRM) and differential interference contrast (DIC) images from time-lapse experiments (see supplemental Movies S1–S4) at 30 min postseeding show strong adhesions (*dark areas* in cells indicated by *red arrowheads*) in the majority of parental RPE-1 cells (labeled as WT). *ADAMTS9* KO RPE-1 cells (labeled as D12) also attach, but only occasional cells form strong adhesions at this early point. *B*, IRM of parental RPE-1 (labeled as WT) and *ADAMTS9* KO (D12) cells 24 h postseeding from time-lapse images showing poorly formed fibrillar adhesions in D12 cells (contrasting with discrete and peripheral fibrillar adhesions in parental RPE-1 cells (*red arrowheads*)). D12 cells show trailing edge filamentous extensions (*blue arrowheads*), which are lacking in parental RPE-1 cells. The *arrow* indicates the direction of cell migration, which was determined by live imaging. *C*, distribution of IRM pixel intensities comparing parental RPE-1 cells (Wt, *blu*e) and D12 cells (*red*). *Dotted lines bin dark* (0–85), *gray* (85–170), and *bright* (170–256) pixels. *D*, violin plot of binned pixel intensities per cell shows that D12 cells have fewer dark pixels (strong focal adhesions). N = 20 cells per group, ∗∗∗*p* < 0.0005, two-tailed unpaired Student *t* test. The scale bars represent 25 μm in (*A*) and 20 μm in (*B*). ADAMTS, a disintegrin-like and metalloproteinase domain with thrombospondin type 1 repeats.

Prior work had showed accumulation of ADAMTS9 substrates versican and fibronectin in the ECM of *ADAMTS9* mutant cells when compared to the parental RPE-1 cells (11, 35). Both substrates are relevant to cell adhesion, with versican being antiadhesive (39, 40, 41) and fibronectin being proadhesive (42, 43). We therefore compared RPE-1 and D12 morphology and adhesion using interference reflection microscopy (IRM), an antibody-independent optical technique for visualizing cell–substratum contacts on a glass surface (44), coupled with differential interference contrast microscopy. In IRM images, cellular regions closely adherent to the substratum appear dark, whereas those further away from the substratum provide a brighter signal (45). Thirty minutes after seeding, WT RPE-1 cells assumed both rounded and slightly flattened shapes and many showed dark central areas on IRM corresponding to the formation of initial adhesions with the glass substratum (Fig. 1*A* and supplemental Movie S1). At this time point, D12 cells had also attached but showed little adhesion on IRM (Fig. 1*A* and supplemental Movie S2). After 24 h, parental RPE-1 cells had robust fibrillar adhesions represented by discrete dark streaks, whereas D12 cells specifically showed a reduction in leading edge fibrillar adhesions, with an increase in leading edge close contacts (gray areas). D12 cells had filamentous protrusions at their trailing edges rarely seen in parental RPE-1 cells (Fig. 1*B* and supplemental Movie S3). Quantitation of pixel intensity showed statistically significant reduction of dark pixels under D12 cells, suggestive of reduced focal/fibrillar adhesions (Fig. 1, *C* and *D*). The differences in the cell–matrix interface observed at both 30 min and 24 h, and lack of primary cilia in D12 cells as previously reported (11), prompted consideration of cell surface and transmembrane molecules as ADAMTS9 substrates. This was addressed by degradomic comparison of parental RPE-1 and D12 cells and their conditioned medium.

### TAILS Identifies Degradome Alterations in the Medium of ADAMTS9-Deficient cells

**Fig. 2:**
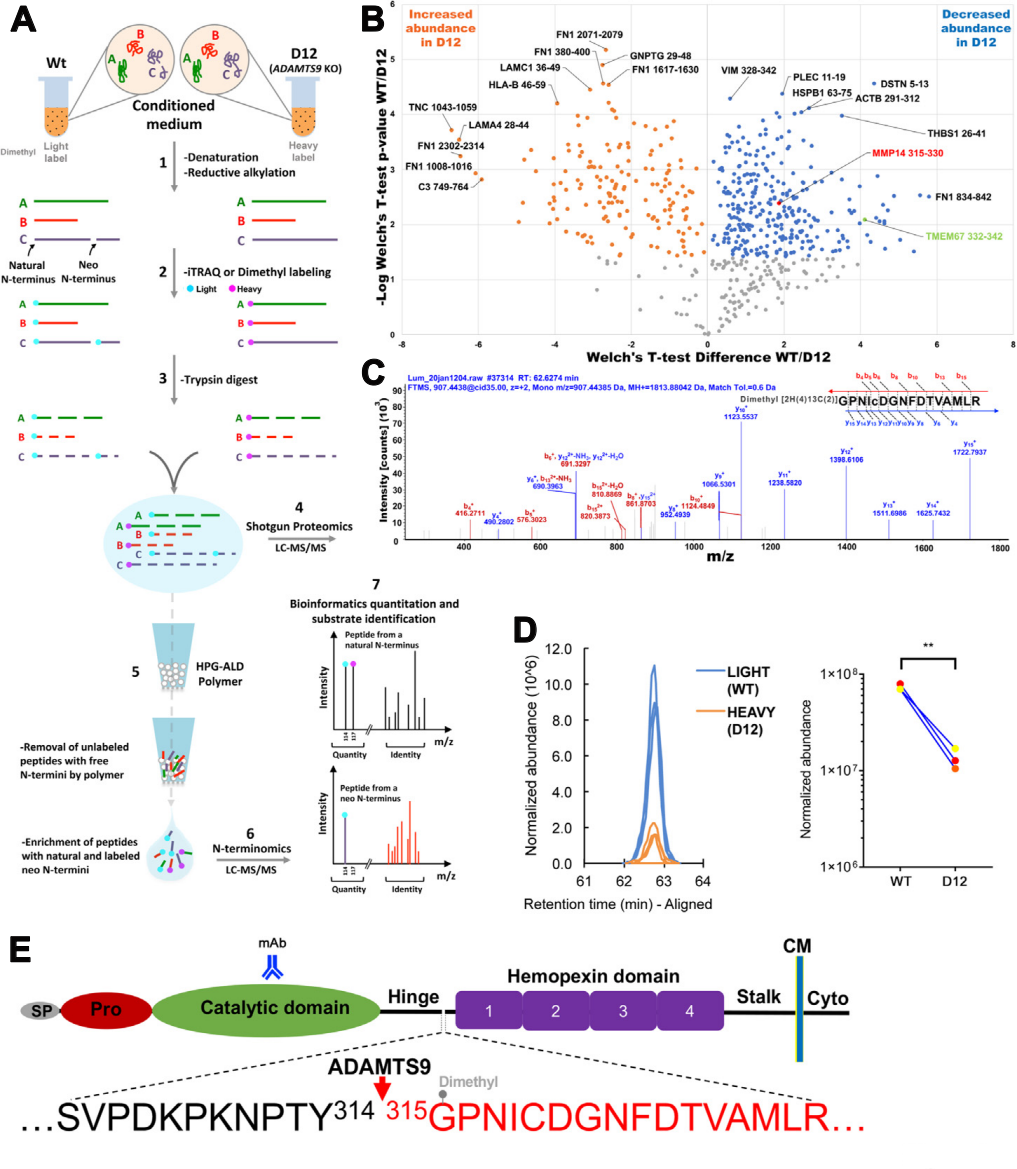
**Identification of MT1-MMP as a novel ADAMTS9 substrate.***A*, schematic illustrating the key experimental steps of the TAILS (terminal amine isotopic labeling of substrates) strategy as applied to parental RPE-1 cells (Wt) and D12 conditioned medium (using reductive dimethylation of protein N termini) and to cell lysates (using iTRAQ labeling of protein N termini). *B*, volcano plot of neo-N terminal peptides arising from secreted and cell-surface proteins identified by TAILS of the medium. Peptides indicating MT1-MMP cleavage at Y^314^-G^315^ and TMEM67 cleavage at K^322^-F^333^ are identified by *red* and *green colors,* respectively and show a statistically significant reduction in D12 cells. *C*, MS2 profile of the MT1-MMP peptide (GPNICDGNFDTVAMLR), which was dimethyl labeled at the N terminus and reduced in the medium of D12 cells. *D*, extracted ion chromatograms of the MT1-MMP precursor ion from triplicate experiments (*left*) and quantitation of normalized abundance of the identified MT1-MMP neo-peptide in triplicate experiments (*right*). ∗∗*p* < 0.005, two-tailed unpaired Student *t* test. *E*, cartoon of MT1-MMP domain structure indicating the location within the hinge of the ADAMTS9 cleavage site deduced from the peptide in (*C*). The four blades of the hemopexin domain β-propeller structure are numbered in sequence from N terminus to C terminus. ADAMTS, a disintegrin-like and metalloproteinase domain with thrombospondin type 1 repeats; CM, cell membrane; Cyto, cytoplasmic tail; mAb, mAb to catalytic domain; MS, mass spectrometry; MT-MMP, membrane type-matrix metalloproteinase; Pro, propeptide; SP, signal peptide.

Parental RPE-1 and D12 cells were cultured in serum-free medium which was analyzed using a TAILS workflow performed in triplicate, in which we employed duplex reductive dimethylation with stable light and heavy isotopes of formaldehyde for labeling proteins from the respective samples (Fig. 2*A*). LC-MS/MS of the differentially labeled proteins, which were combined for the analysis to eliminate run-to-run variation and for precise relative quantitation, identified numerous high-confidence dimethyl-labeled N-terminal peptides with high labeling efficiency (supplemental Table S1 and supplemental Fig. S1). Peptides representing N termini of intact proteins or protein fragments were identified by an N-terminal dimethyl label or other blocking entity. Because of the intentional enrichment of blocked N termini in TAILS and inclusion of the search parameter “semitryptic,” an expected bias toward N-terminal modifications such as pyro-Glu and acetylation and blocked tryptic-like N termini was observed. The removal and inactivation of dimethylation reagents at the conclusion of labeling in the experimental workflow precludes the possibility of generating tryptic peptides experimentally, but the numerous N-terminally labeled peptides observed to have an upstream Arg residue (supplemental Data File) suggest the presence of trypsin-like proteases or other proteases with Arg-C preference in the cultures.

Positional annotation of the peptides determined which originated internal to constitutive processing sites such as signal peptide or propeptide excision. Subsequently, statistical analysis of their relative abundance based on aligned, normalized extracted ion chromatograms of the light/heavy MS1 peaks identified several internally originating peptides (neo-N terminal peptides), arising from secreted and transmembrane proteins with significantly different abundance (Fig. 2*B*). Among neo-N terminal peptides with reduced abundance in the medium of D12 cells, and thus potentially indicative of ADAMTS9 substrates, were six peptides from the fibronectin (a known substrate), a peptide from the cilium transition zone transmembrane protein Tmem67 and an MT1-MMP (MMP14) peptide, G^315^PNICDGNFDTVAMLR (Fig. 2, *B*–*D* and supplemental File S1). The position of this N-terminally labeled MT1-MMP peptide suggested cleavage at the Tyr^314^-Gly^315^ peptide bond, located in the “hinge” between the MT1-MMP catalytic domain and hemopexin domain (Fig. 2*E*). Consistent with focus on identifying ADAMTS9 substrates, we did not further consider N termini that increased in the absence of the protease, since there could be alternative explanations for their occurrence including transcriptional changes occurring well downstream of the direct impact of ADAMTS9, MT1-MMP, or other substrates.

**Fig. 3:**
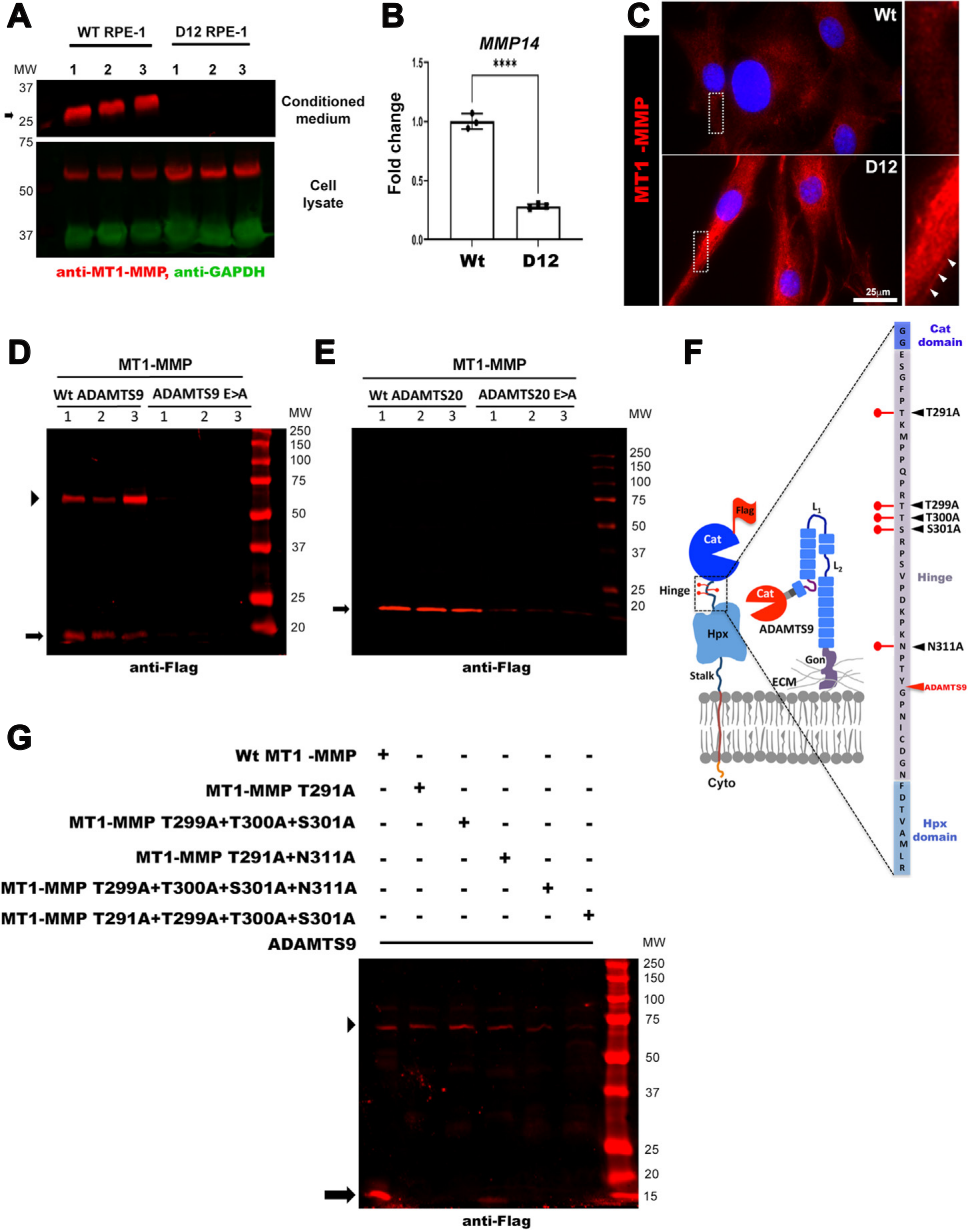
**MT1-MMP is cleaved by ADAMTS9 and ADAMTS20, and MT1-MMP hinge glycosylation is essential for proteolysis by ADAMTS9.***A*, Western blot of parental RPE-1 (Wt) and D12 conditioned medium and cell lysate with a catalytic domain-specific antibody and anti-GAPDH as control showing release of the MT1-MMP catalytic domain (*arrow* indicates the catalytic domain fragment) in Wt but not D12 cells and corresponding to this, higher MT1-MMP signal in D12 lysates. *B*, quantitative RT-PCR analysis of *MMP14* (MT1-MMP) transcript levels in parental RPE-1 (Wt) and D12 cells shows significantly downregulated expression in D12 cells. Error bars indicate S.D., ∗∗∗∗*p* < 0.0001, two-tailed unpaired Student *t* test. *C*, cell surface immunostaining of MT1-MMP utilizing a catalytic domain antibody (*red*) shows more intense MT1-MMP staining in D12 cells. Nuclei are stained *blue* by DAPI. Molecular weight markers are shown on the *right* of the blot. High-magnification areas shown on the *right* are marked by the *white boxes*. *White arrowheads* indicate the cell membrane. The scale bar represents 25 μm. *D*, Western blot (anti-FLAG) of the medium of HEK293T cells cotransfected in triplicate with MT1-MMP and ADAMTS9 showing molecular species of ∼60 kDa (*arrowhea*d), corresponding to the MT1-MMP ectodomain and presumed to occur within the MT1-MMP stalk and ∼15 kDa (*arrow*), which are lacking in the medium of cells cotransfected with catalytically inactive ADAMTS9 (ADAMTS9E>A). Molecular weight markers are shown on the *righ*t of the blot adjoining the protein ladder. *E*, Western blot (anti-FLAG) of the medium of HEK293T cells cotransfected in triplicate with MT1-MMP and ADAMTS20 showing a single 15 kDa fragment (*arrow*), which is lacking in the medium of cells cotransfected with catalytically inactive ADAMTS20 (ADAMTS20E>A). Molecular weight markers are shown on the *right* of the blot. *F*, schematic illustrating the MT1-MMP hinge in greater detail and depicting a model of ADAMTS9 cleavage at the MT1-MMP hinge. The hinge sequence is shown at *far right* flanked by the catalytic (Cat) domain and hemopexin (Hpx) domains. Lollipops indicate the glycosylated residues, and *black arrowheads* indicate the specific mutations at these sites. The ADAMTS9 cleavage site is indicated by the *red arrowhead*. *G*, Western blot of the medium from HEK293T cells cotransfected with ADAMTS9 and MT1-MMP or the indicated glycosylation mutants. Note the absence of the ∼15 kDa fragment indicated by the *arrow* in all glycosylation mutants and additionally, lack of stalk cleavage in the T291A + T299A + T300A + T301A mutant (∼60 kDa fragment indicated by *arrowhead*). Molecular weight markers are shown on the *right* of the blot. ADAMTS, a disintegrin-like and metalloproteinase domain with thrombospondin type 1 repeats; Cyto, cytoplasmic tail; ECM, extracellular matrix; Gon, Gon-1 domain; MT-MMP, membrane type-matrix metalloproteinase.

In agreement with the possibility that ADAMTS9 shed the MT1-MMP ectodomain from the cell surface, Western blotting under reducing conditions demonstrated a ∼35 kDa species reactive with a mAb against the MT1-MMP catalytic domain in the medium of parental RPE-1 cells, but not D12 cells. (Fig. 3*A* and supplemental Fig. S2). Moreover, Western blot showed greater MT1-MMP levels in the D12 lysate (Fig. 3*A*). However, RT-qPCR also disclosed a statistically significant reduction in MT1-MMP (MMP14) mRNA in the D12 cells (Fig. 3*B*), prompting analysis of whether the TAILS neo-N terminal peptide reflected reduced cleavage or reduced MT1-MMP abundance in D12 cells. Staining of RPE-1 and D12 cells without permeabilization was undertaken, which illustrated more intense MT1-MMP staining on the surface of D12 cells (Fig. 3*C*) consistent with greater MT1-MMP observed by Western blotting in D12 lysate (Fig. 3*A*). Furthermore, when ADAMTS9 was co-expressed with Flag-tagged MT1-MMP in HEK293T cells, Western blotting under reducing conditions demonstrated an ∼15 kDa anti-Flag–reactive species as well as an additional ∼60 kDa species, both of which were absent when the catalytically inactive mutant, ADAMTS9-EA was co-expressed (Fig. 3*D*). Co-expression of ADAMTS20 with Flag-tagged MT1-MMP in HEK293T cells demonstrated the presence of a 15 kDa, anti-Flag–reactive species but not the 60 kDa species in the medium, whereas neither were seen when catalytically inactive ADAMTS20 was co-expressed (Fig. 3*E*). Although the precise ADAMTS20 cleavage site is unknown, the size of the catalytic domain fragment released into the medium suggested that ADAMTS20 cleaves MT1-MMP similarly to ADAMTS9 (Fig. 3, *D* and *E*). The additional 60 kDa anti-Flag–reactive species seen upon ADAMTS9 overexpression (Fig. 3*D*) suggested the possibility of juxta-membrane cleavage in the MT1-MMP stalk (Fig. 3*F*).

### *O*-Glycosylation of the MT1-MMP Hinge Domain is Required for ADAMTS9-Mediated Catalytic Domain Shedding

The MT1-MMP hinge contains potential sites for *O-*glycosylation at Thr^291^, Thr^299^, Thr^300^, and Ser^301^ and one potential site for N-glycosylation at Asn^311^(Fig. 3*F*), although Pro at the Xaa position in the consensus N-glycosylation sequence, Asn-Xaa-Ser/Thr, suggests that this modification may be lacking (46). *O-*glycosylation of the MT1-MMP hinge protects it from autolysis (47), whereas its loss affects neither cell surface presentation nor dimerization but results in a closed catalytic domain pocket inaccessible for interaction with TIMP-2, which directly binds to and recruits pro-MMP2 for activation (34). We therefore inquired if MT1-MMP *O*-glycosylation similarly affected its proteolysis by ADAMTS9. Cotransfection of ADAMTS9 with WT or mutant flag-tagged MT1-MMP–carrying mutations of glycosylated sites (Thr^291^Ala, Thr^299^Ala, Thr^300^Ala, Ser^301^Ala, Asn^311^Ala) showed reduced proteolysis at the hinge, although cleavage at the presumed C-terminal site that generated the 60 kDa fragment was relatively unaffected (Fig. 3*G*). Quadruple combined mutations of *O*-glycosylated sites (Thr^291^Ala + Thr^299^Ala + Thr^300^Ala + Ser^301^Ala + Asn^311^Ala) led to loss of both the ∼15 kDa and ∼60 kDa bands (Fig. 3*G*). These results suggest that *O*-glycosylation of the MT1-MMP hinge is necessary for ADAMTS9-mediated proteolysis of MT1-MMP.

### Degradomic Identification of a Second ADAMTS9 Cleavage Site in the MT1-MMP Hemopexin Domain

Since MT1-MMP is a type 1 transmembrane proteins, detection of a neo-N terminal peptide in the conditioned medium implied occurrence of one or more additional cleavages downstream of Tyr^314^-Gly^315^ that released the MT1-MMP ectodomain into the medium. At least one such cleavage was suggested by the 60 kDa fragment detected upon cotransfection of MT1-MMP and ADAMTS9. We investigated the possibility of additional cleavage sites by targeted searches of the TAILS and pre-TAILS datasets from the medium (supplemental Files S2 and S3) for additional MT1-MMP peptides and 8-plex iTRAQ-TAILS comparison of RPE-1 and D12 cell lysate.

**Fig. 4:**
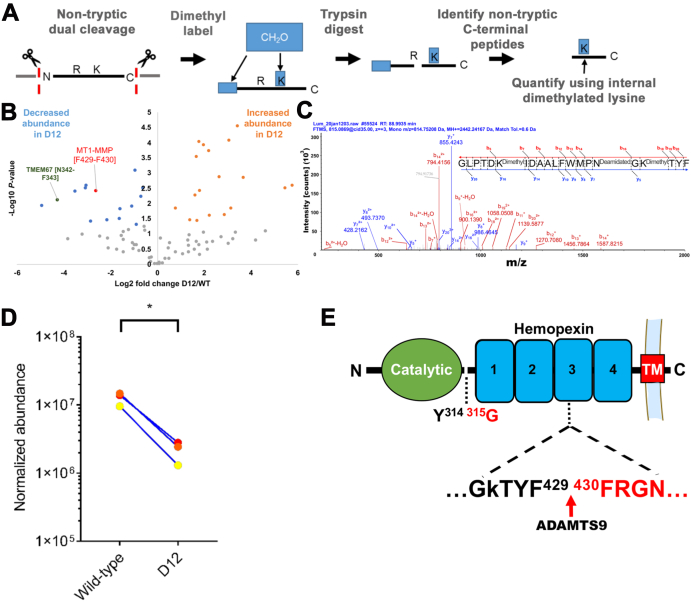
**Degradomics identification of a cleavage site in the MT1-MMP hemopexin domain.***A*, schematic of the strategy for identifying nontryptic peptides in the medium of parental RPE-1 cells and D12 cells and quantitation *via* internal dimethyl-labeled lysine residues. *B*, volcano plot of peptides identified with this strategy highlighting MT1-MMP and TMEM67 peptides and indicating cleavages at Phe^429^-Phe^430^ (*red*) and Asn^342^-Phe^343^ (*dark blue*), respectively. *C*, MS2 profile of the MT1-MMP peptide GLPTDKIDAALFWMPNGKTYF. *D*, quantitation of normalized abundance of the identified MT1-MMP peptide in triplicate experiments. ∗*p* < 0.05, two-tailed unpaired Student *t* test. *E*, location of the deduced cleavage site in blade 3 of the hemopexin domain. The hinge cleavage site is also shown. MS, mass spectrometry; MT-MMP, membrane type-matrix metalloproteinase; TM, transmembrane segment.

We first analyzed TAILs and pre-TAILS data from the medium for differentially abundant MT1-MMP peptides with nontryptic C termini that could indicate a cleavage site (Fig. 4*A*). This strategy identified two peptide spectrum matches for the peptide **GLPTDKIDAALFWMPNGKTYF**^429^↓(F^430^) (MS-identified sequence in bold letters, flanking amino acid in parentheses, with the residues at the nontryptic site numbered) (Fig. 4, *B*–*D* and supplemental Fig. S3). This peptide was tryptic (preceded by Arg) at its N terminus, which was not dimethyl-labeled and nontryptic at its C terminus and was located within the third blade of the four-bladed β-propeller structure of the hemopexin domain. The peptide was quantifiable from dimethyl labeling of two internal Lys residues and was more abundant in WT cells (Fig. 4, *C* and *D*). Cleavage at the F^429^-F^430^ site is unlikely to partition MT1-MMP into fragments because of the disulfide bond between the first and fourth blades of the β-propeller, although it could modify the tertiary structure of the hemopexin blade, β-propeller, or full-length MT1-MMP (Fig. 4*E*).

Among differentially abundant iTRAQ-labeled MT1-MMP peptides identified in RPE-1 and D12 cell lysates (supplemental File S4), the only informative differentially abundant neo-N terminal peptide had the sequence (Thr^313^) ↓**Y**^**314**^ **GNICDGNFDTVAMLR** (MS-identified peptide sequence in bold letters and flanking amino acid in parentheses), suggesting cleavage at Thr^313^-Tyr^314^ (supplemental File S4, *A* and *B*). This peptide was more abundant in the D12 cell lysate, suggesting a cleavage that occurred at reduced levels in the presence of ADAMTS9 (supplemental File S4*C*).

### ADAMTS9 Knockout is Associated With Increased MMP2 Zymogen Activation and Enhanced Collagenase Activity

**Fig. 5:**
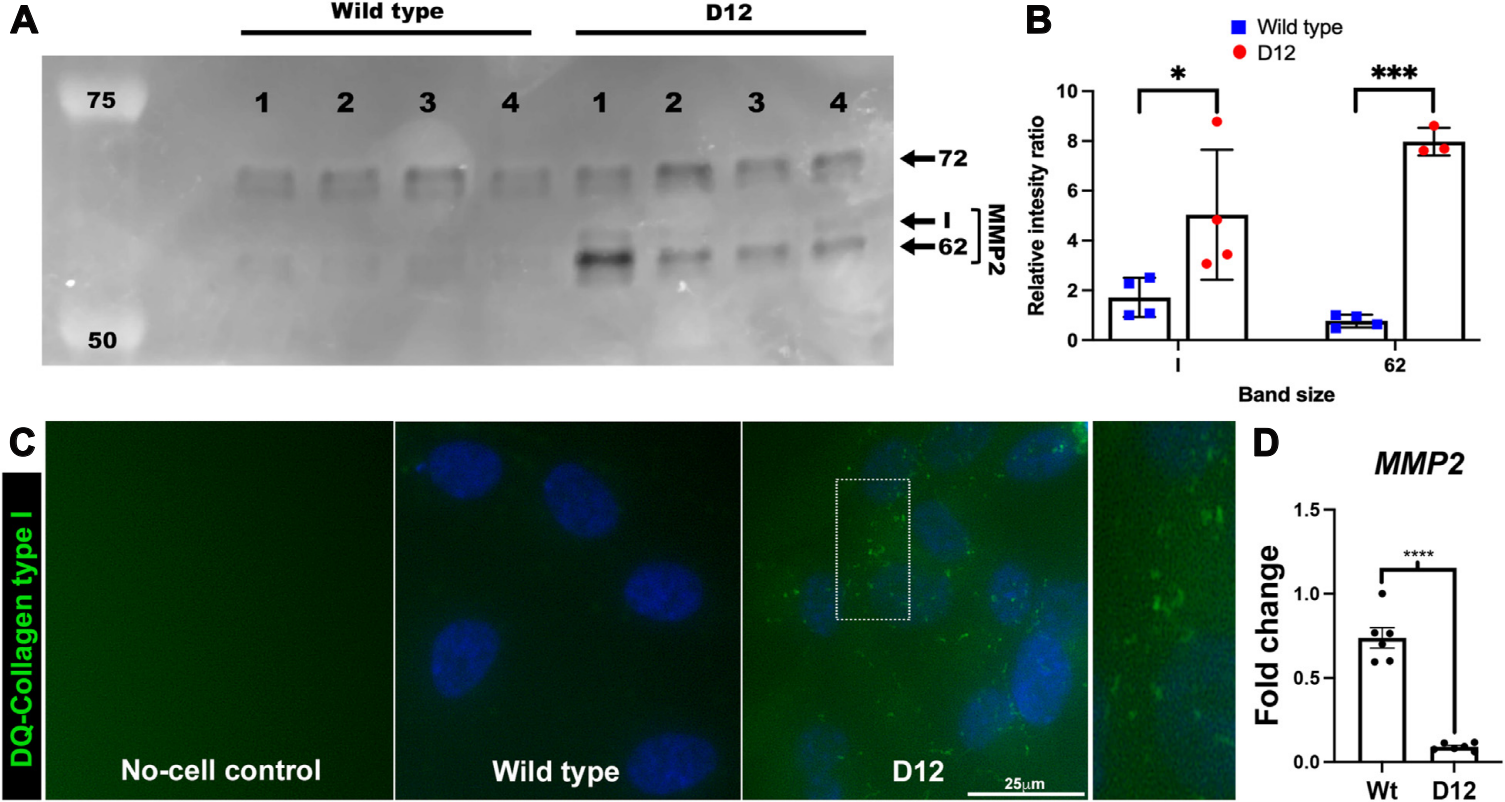
**Increased pro-MMP2 activation and collagenase activity in D12 cells as a result of increased cell-surface MT1-MMP.***A*, gelatin zymography of the medium from parental RPE-1 cells (WT) and D12 cells shows increased active MMP2 (62 kDa) as well as an intermediate species (*I*) in the D12 medium. *B*, quantitation of band intensities from (*A*) shows significantly higher active MMP2 levels in the D12 medium. N = 4, each group, error bars indicate SD, ∗ indicates *p* < 0.05, ∗∗∗ indicates *p* < 0.05, Student *t* test. *C*, parental RPE-1 cells and D12 cells were cultured on DQ collagen type I–coated plates for 24 h, and areas of collagenolysis were visualized by *green* fluorescence, showing greater activity in D12 cells than parental RPE-1 cells. *D*, quantitative RT-PCR analysis shows reduced *MMP2* mRNA in D12 cells. Error bars indicate SD, ∗∗∗∗*p* < 0.0001, two-tailed unpaired Student *t* test. The scale bar in (*C*) represents 25 μm. MT-MMP, membrane type-matrix metalloproteinase.

Cell surface MT1-MMP is an activator of pro-MMP2 (32). Gelatin zymography of conditioned medium from parental RPE-1 cells and D12 cells showed increased pro-MMP2 activation (*i.e.,* more of the 62 kDa active MMP-2, as well as a 65 kDa intermediate form) in the absence of ADAMTS9 (Fig. 5, *A* and *B*). Culture of parental RPE-1 and D12 cells on DQ-collagen I–coated slides showed greater proteolysis beneath D12 cells, revealed by fluorescence dequenching upon substrate proteolysis (Fig. 5*C*). Thus, ADAMTS9 potentially regulates both MT1-MMP activity on the cell surface and downstream MMP2 activation. Increased MMP2 activity noted on zymograms was not a consequence of higher *MMP2* mRNA, which in fact was significantly lower in the D12 cells (Fig. 5*D*).

### MT1-MMP Modulates Cilium Length in RPE-1 Cells Without Affecting Cilium Biogenesis

**Fig. 6:**
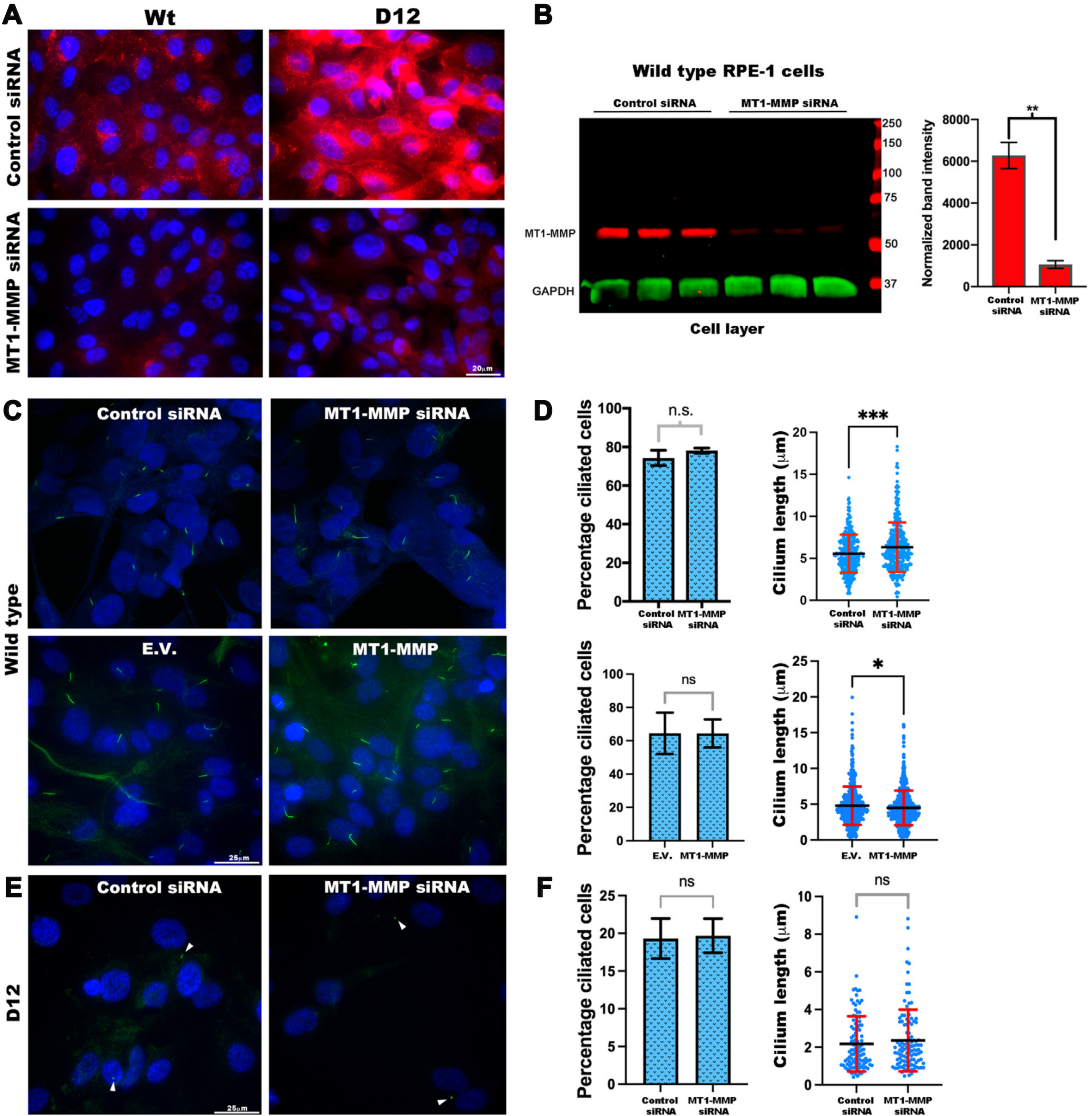
**Depletion or overexpression of cellular MT1-MMP affects cilium length but not cilium biogenesis.***A*, immunostaining of cell surface MT1-MMP (*red*) shows that *MMP14* siRNA effectively depleted MT1-MMP in both parental RPE-1 cells (Wt) and ADAMTS9-edited RPE-1 D12 cells. *B*, Western blot of parental RPE-1 cells treated with control siRNA or *MMP14* siRNA shows significant depletion of MT1-MMP (*red*) normalized to GAPDH (*green*) as a measure of knockdown. Error bars indicate SD, ∗∗ indicates *p* < 0.005, two-tailed unpaired Student *t* test analysis. *C* and *D*, primary cilium staining using acetylated α-tubulin antibody (*green*) in parental RPE-1 cells treated with control siRNA and *MMP14* siRNA (*upper panels*) or transfected with MT1-MMP or empty vector (E.V.) (*lower panels*) showed that MT1-MMP depletion did not affect the percentage of ciliated cells but slightly increased cilium length. MT1-MMP overexpression also did not affect the percentage of ciliated cells but slightly decreased cilium length. *Red error bars* indicate SD, *black lines* indicate mean, ∗∗∗*p* < 0.0005, ∗*p* < 0.05 two-tailed unpaired Student *t* test. *E* and *F*, primary cilium analysis using acetylated α-tubulin immunostaining (*green*) after depletion of MT1-MMP in D12 cells showed no impact on the percentage of ciliated cells nor cilium length. *Red error bars* indicate SD, *black lines* indicate the mean. The scale bars represent 20 μm in (*A*) and 25 μm in (*B*). ADAMTS, a disintegrin-like and metalloproteinase domain with thrombospondin type 1 repeats; MT-MMP, membrane type-matrix metalloproteinase.

ADAMTS9 is required for primary cilium biogenesis in humans and in mice, that is, the primary cilium either fails to form in cells of *Adamts9* or *Adamts9+Adamts20* mutant mice *in vivo* and in D12 cells, or if formed, is extremely short (11, 24). We therefore inquired if altered cell surface MT1-MMP levels affected ciliogenesis in parental RPE-1 cells. First, using an siRNA which provided substantial MT1-MMP depletion (Fig. 6, *A* and *B*), we observed no impact on the percentage of RPE-1 cells with primary cilia after serum starvation, although cilium length was slightly increased compared to cells transfected with control siRNA (Fig. 6, *C* and *D*). Overexpression of MT1-MMP in parental RPE-1 cells did not alter the percentage of ciliated cells either and resulted in slightly shorter primary cilia than cells transfected with the empty vector (Fig. 6, *C* and *D*). Thus, increasing or decreasing MT1-MMP had no impact on ciliogenesis, although a subtle effect on cilium length was observed. To inquire if loss of cilia in the absence of ADAMTS9 could be explained by increased MT1-MMP activity, we depleted MT1-MMP in D12 cells and induced ciliogenesis by serum starvation. Although MT1-MMP knockdown led to even lower levels than in parental RPE-1 cells as judged by immunofluorescence of unpermeabilized cells (lower panels, Fig. 6*A*), neither the percentage of ciliated cells nor cilium length were affected (Fig. 6, *E* and *F*). These results indicate that lack of cilium biogenesis in the absence of ADAMTS9 was not due to increased cell surface MT1-MMP or greater MMP2 activation.

### Reversal of Reduced Adhesion in ADAMTS9-Deficient D12 Cells by MT1-MMP Knockdown

**Fig. 7:**
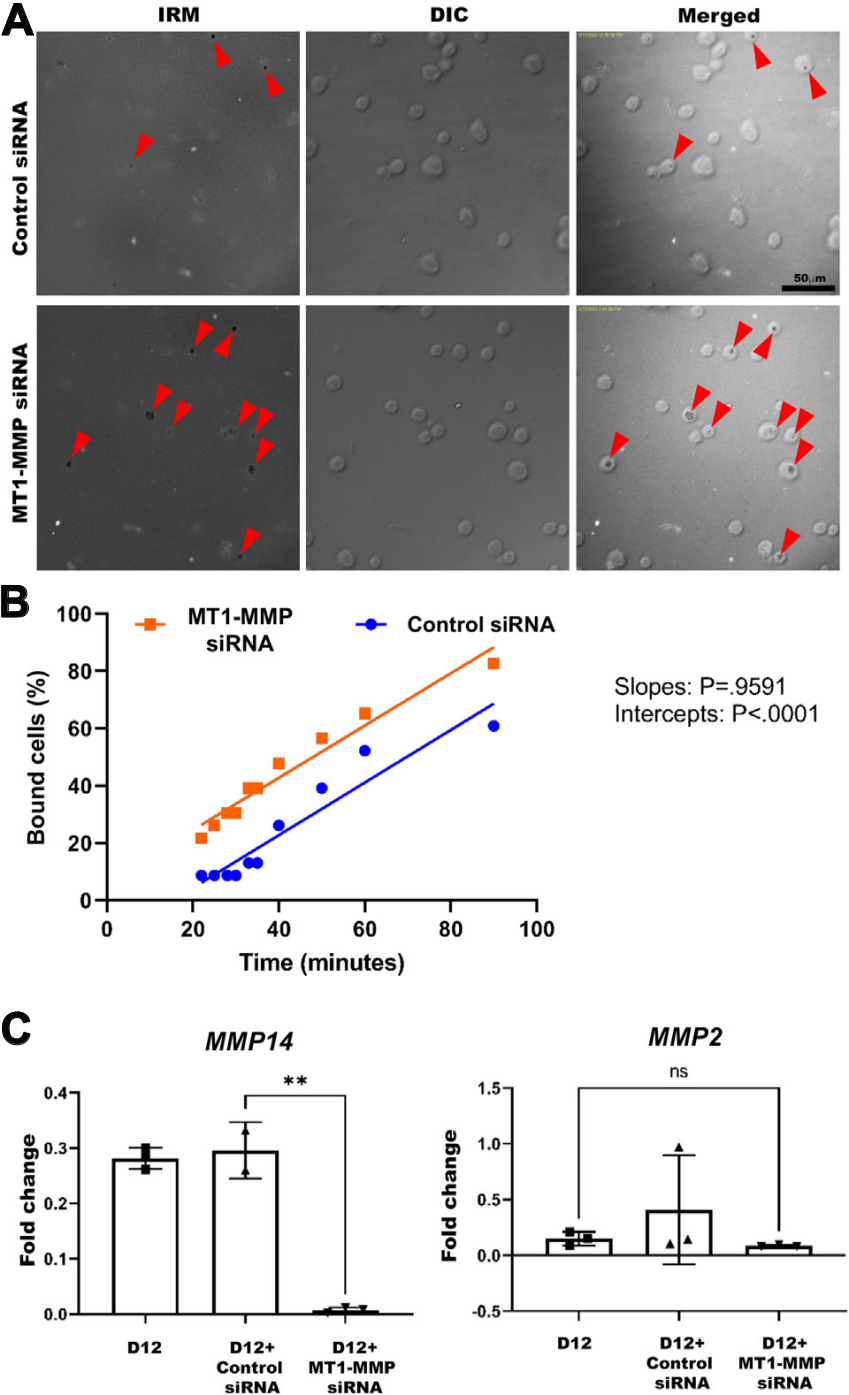
**MT1-MMP depletion restored cell–substratum interaction in RPE1-ADAMTS9**^**KO**^**cells.***A*, still images taken from concurrent, aligned interference reflection microscopy (IRM) and differential interference contrast (DIC) time-lapse imaging show reversion of the cell–substratum interface to that noted at the same time point in parental RPE-1 cells (see Fig. 1*A*) after *MMP14* knockdown. *Red arrowheads* indicate attached cells with dark contacts. *B*, percentage of D12 cells bound by focal adhesions observed by time-lapse microscopy after transfection with MT1-MMP siRNA (*orange*) and scrambled siRNA (*blue*) shows consistently better cell attachment in the former. *C*, quantitative RT-PCR analysis for *MMP14* and *MMP2* transcripts in D12 cells treated with control siRNA and *MMP14* siRNA shows effective knockdown and suggests that stronger attachment after *MMP14* knockdown is not due to altered transcription of MMP2. Error bars indicate SD, ∗∗ indicates *p* < 0.0005 in two-tailed unpaired Student *t* test. The scale bar represents 50 μm in (*A*). ADAMTS, a disintegrin-like and metalloproteinase domain with thrombospondin type 1 repeats; MT-MMP, membrane type-matrix metalloproteinase.

**Fig. 8:**
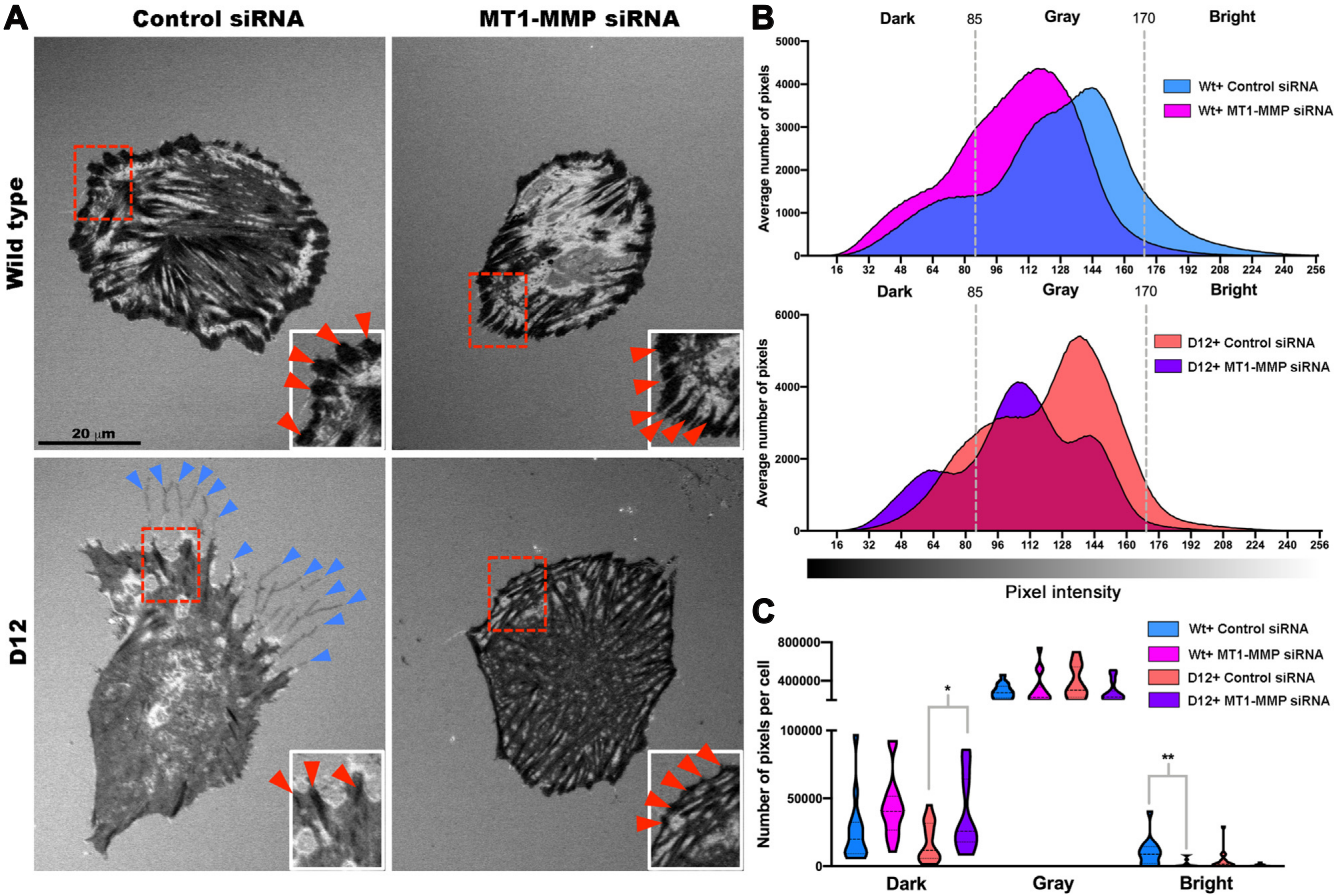
**MT1-MMP knockdown improves focal adhesion formation in D12 cells.***A*, interference reflection microscopy (IRM) imaging 24 h after seeding of parental RPE-1 cells and D12 cells treated with control siRNA or *MMP14* siRNA shows an increase in fibrillar adhesions, especially at the cell periphery (*red arrowheads*, inset box) and lack of trailing edge filamentous extensions observed in D12 cells (*blue arrowheads*). *B*, distribution of IRM pixel intensities comparing parental RPE-1 cells treated with control siRNA (*blue*) or MT1-MMP siRNA (*pink*) and D12 cells treated with control siRNA (*salmon*) or MT1-MMP siRNA (*purple*). *Dashed lines* indicate binning of *dark* (0–85), *gray* (85–170), and *bright* (170–256) pixels. *C*, violin plot of binned pixel intensities per cell showing increased dark pixels in D12 cells treated with MT1-MMP siRNA compared to control siRNA treatment and decreased bright pixels in WT cells treated with MT1-MMP siRNA. N = 14 to 16 cells per treatment group, ∗*p* < 0.05, ∗∗*p* < 0.005, two-tailed unpaired Student *t* test. The scale bar represents 20 μm in (*A*). MT-MMP, membrane type-matrix metalloproteinase.

To inquire if impaired adhesion in D12 cells (Fig. 1*A*) was due to increased MT1-MMP activity, we repeated IRM after MT1-MMP knockdown. Time-lapse IRM imaging demonstrated accelerated emergence of focal adhesions 30 min after seeding D12 cells treated with *MMP14* siRNA compared to control siRNA treatment (Fig. 7, *A* and *B*). In these cells, *MMP14* siRNA was clearly effective in reducing its expression, whereas there was no impact on *MMP2* expression (Fig. 7*C*). Twenty-four hours postseeding, both parental RPE-1 and D12 cells with *MMP14* knockdown demonstrated a shift in the relative proportion of close contacts (gray) and focal/fibrillar adhesions (dark areas), with increased focal adhesions evident in the D12 cells in response to MT1-MMP depletion (Fig. 8, *A* and *B*). Trailing edge membrane protrusions observed in D12 cells were only occasionally observed after MT1-MMP depletion (Fig. 8*A*; supplemental Movies S3 and S4). Quantification of binned pixel intensities in the spectrum of dark to bright pixels per cell demonstrated a statistically significant gain of dark pixels in D12 cells in response to MT1-MMP depletion, whereas WT RPE-1 cells exhibited a statistically significant reduction of the bright pixels and a moderate increase of the darker pixels, albeit statistically insignificant (Fig. 8*C*).

## Discussion

The present work shows that RPE-1 cells lacking ADAMTS9 delay initiation of focal adhesions and form fewer peripheral fibrillar adhesions with a glass substratum than parental RPE-1 cells. A degradomic analysis of these cells, which was undertaken to define the proteolytic changes possibly associated with this effect, identified several potential substrates. Here, we undertook detailed validation and further analysis of MT1-MMP, since this cell surface protease is a major participant in ECM proteolysis, both directly and *via* activation of other proteases (48, 49). Like ADAMTS9 and ADAMTS20, MT1-MMP is strongly implicated in embryonic development (50, 51, 52, 53), but correspondence of *Mmp14* and *Adamts9* phenotypes is not expected since ADAMTS9 mutation leads to increased MT1-MMP, for which no genetic model presently exists. In contrast to *Adamts9*, whose functions span several developmental stages and processes from gastrulation to birth, *Mmp14-*deficient phenotypes substantially reflect its requirement for fibrillar collagen breakdown in the postnatal and juvenile period (50, 54). *Adamts9* null mice do not survive past gastrulation (14), and *Adamts9* and *Adamts20* mRNAs are co-expressed in many tissues so that it may be challenging to determine whether MT1-MMP activity is increased in embryos lacking ADAMTS9 and ADAMTS20.

Most endopeptidases have a selective, albeit broad substrate repertoire, and it is only the rare protease which has a private substrate (*e.g.,* ADAMTS13 appears to cleave only von Willebrand factor). The first identified ADAMTS9 substrates were the aggregating ECM proteoglycans aggrecan and versican (7). TAILS analysis of skin from 1-week-old WT and an *Adamts9* mutant (*Adamts9*^Und4/+^ (30)) identified several ECM components as potential substrates, although none were orthogonally validated. The yeast two-hybrid system was used to screen for ADAMTS9-binding partners, identifying the ECM glycoprotein fibronectin as a potential substrate (55). Proteomics analysis of fibronectin semitryptic peptides in the presence of catalytically active ADAMTS9 identified the cleavage site, and ADAMTS9 was shown to impair fibronectin fibril assembly by cultured fibroblasts (55). The present work, using TAILS and a different cellular system, has identified additional fibronectin cleavages, although not the previously identified site. Thus, ADAMTS9 cleaves three orthogonally validated ECM substrates—versican, aggrecan, and fibronectin and very likely, many others—such that ADAMTS9-dependent phenotypes could have complex mechanistic basis involving failure to cleave multiple substrates.

Impaired adhesion of D12 cells is consistent with prior work, showing that cultured human myometrial smooth muscle cells (SMCs) did not form fibrillar adhesions and were poorly adherent after *ADAMTS9* knockdown (13). Primary myometrial SMCs appear to depend on focal adhesions for their survival, since they underwent anoikis after *ADAMTS9* knockdown (13). However, the CRISPR-Cas9 generated D12 clonal derivative of RPE-1 cells (an immortalized cell line), survived in culture despite a complete lack of ADAMTS9 (11). Versican is evidently a key antiadhesive ADAMTS9 substrate in human myometrial SMCs, since their adhesion was restored by versican knockdown concomitantly with ADAMTS9 knockdown (13). Versican, which also accumulates in the ECM of D12 cells when cultured for 72 h or longer (11, 35), is likely also relevant to altered adhesion of D12 cells, although this possibility was not specifically investigated here. MT1-MMP knockdown had a restorative effect on fibrillar adhesion formation by D12 cells, suggesting that the complex tissue and cell phenotypes resulting from ADAMTS9 and ADAMTS20 deficiency could reflect impaired cleavage of more than one substrate, that is, MT1-MMP, versican, fibronectin, and potentially others.

Sequence analysis of cleavage sites identified in comparative TAILS of WT and *Adamts9*^Und4/+^ skin suggested a preference for aromatic residues Tyr and Phe or the hydrophobic residue Leu at the P1 position (according to the nomenclature of Schechter and Berger) and Thr at P2. The two cleavage sites attributed to ADAMTS9 in MT1-MMP are consistent with this preference, with Tyr and Phe at the P1 position and Gly or Phe, respectively, at P1'. The processing event in the MT1-MMP hinge with higher abundance in D12 cells (Thr^313^-Tyr^314^) may be a constitutive cleavage, such as by autolysis, that may occur if prior processing by ADAMTS9 is lacking. MT1-MMP autolysis was previously reported to occur at Gly^284^-Gly^285^ in the hinge, followed by subsequent cleavage at Ala^255^-Ile^256^ (56, 57). It was reported that the residual cell surface–attached ectodomain generates increased cell surface proteolytic activity by impairing endosomal uptake of full-length MT1-MMP, resulting in higher levels of active enzyme at the cell surface (58). This may explain the observed increase in MT1-MMP staining and activity in D12 cells despite reduced *MMP14* transcription. Our findings will add to the known regulatory mechanisms of MT1-MMP (32, 59).

MT1-MMP was previously shown to attenuate integrin clustering in HT1080 cells adherent to fibronectin; in contrast, MT1-MMP inhibition led to the formation of stable adhesions (60, 61). MT1-MMP mediates proteolysis of fibronectin and other substrates at focal adhesions (62), including ARPE-19 cells, which like the RPE-1 cells used in this study, are derived from retinal pigment epithelium. MT1-MMP is localized to focal adhesions *via* interaction with β1-integrin (63), but it remains to be established whether ADAMTS9 or ADAMTS20 interact directly with integrins at the cell surface and whether they cleave MT1-MMP within focal adhesions or elsewhere. The MT-loop, a segment of the catalytic domain of MT1-MMP ((163)PYAYIREG(170)), that is, likely within the putative N-terminal regions released by ADAMTS9/20 from the cell surface, is essential for MT1-MMP promotion of cellular invasion by localization to β1-integrin–rich cell adhesion complexes at the plasma membrane (61), whereas other work has suggested a key role for the cytoplasmic tail and transmembrane domain in β1-integrin regulation (64). MT1-MMP also interacts with α_v_β_3_ integrin, which it proteolytically cleaves (65, 66, 67). Notably, MT1-MMP–deficient myeloid progenitors and mouse lung endothelial cells have larger focal adhesions (68). It was postulated that this effect was potentially related to accumulation of adhesive proteins such as fibronectin and collagen I which are MT1-MMP substrates and a lower turnover of focal adhesions (69). Our data, showing that lack of ADAMTS9/20 led to fewer focal adhesions in RPE1 cells, with greater cell surface collagenase activity and MMP2 activation, appear to be consistent with the expected effect of increased MT1-MMP at the cell surface.

Previously, enzymatic deglycosylation, site-directed mutagenesis, and lectin precipitation assays were used to demonstrate that the MT1-MMP hinge region contains *O-*linked carbohydrates on Thr^291^, Thr^299^, Thr^300^, and/or Ser^301^ (34). MT1-MMP autolysis is increased in the absence of glycosylation (47), and MT1-MMP lacking glycosylation at these sites is stable but unable to bind TIMP-2, preventing formation of the MT1-MMP–TIMP2–pro-MMP2 trimeric complex essential for pro-MMP2 activation (34). The positive effect of MT1-MMP hinge glycosylation on susceptibility to cleavage by ADAMTS9 extends prior work, identifying an influential role for N-linked and *O*-linked glycosylation on the activity of ADAMTS9, MT1-MMP and other proteases (70, 71, 72, 73).

*MMP21* mutations can cause heterotaxy (74, 75, 76), a ciliopathy, but there is no evidence that MT1-MMP is required for the formation or function of primary cilia, which are distinct from motile cilia present on many epithelial cells. *Mmp14* KO mice do however have defective ependymal motile cilia, which are thought to underlie hydrocephalus in these mice (77). We show that MT1-MMP knockdown has no impact on cilium biogenesis, although cilia are slightly longer than in parental RPE-1 cells. Hydrocephalus is also observed in *Adamts20*^Bt/Bt^ mice (78), although its mechanism is yet to be resolved.

The present findings expand the protease web, in which positive and negative feedback loops, including activation or elimination of protease activity, are mediated by proteases within the same or different families (79). For example, MT1-MMP activates MMP-2 and MMP-13 in a feed-forward mechanism, whereas it inactivates the transmembrane protease ADAM9 (80). In contrast to the effect of the transmembrane metalloprotease ADAM12 in activating MT1-MMP *via* a nonproteolytic mechanism that promotes pro-MMP2 activation (81), ADAMTS9 and ADAMTS20 are shown here to cleave MT1-MMP and suppress proMMP-2 activation as a potential physiological role. Although the findings of this study were elucidated exclusively in RPE-1 cells, it is possible that ADAMTS9 and ADAMTS20 may act similarly in tissues where they are expressed alongside MT1-MMP, such as during ocular development and in vascular endothelium or craniofacial mesenchyme. ADAMTS9 is constitutively expressed in endothelial cells in most mouse capillary beds and is antiangiogenic (9), which could be potentially explained by modulation of cell surface MT1-MMP and MMP2 activity, which are strongly pro-angiogenic (53) and by promotion of focal adhesion formation. In this context, genome-wide association studies have associated the *ADAMTS9* locus with age-related macular degeneration (82, 83), a major cause of adult-onset blindness, where RPE cell death and dysregulated angiogenesis are prominent features. In eyes at high risk of age-related macular degeneration, one study identified lower *ADAMTS9* mRNA levels (84), which could lead to higher MT1-MMP activity, and thus greater pro-MMP2 activation, together promoting retinal angiogenesis. In future work, it will be important to apply the findings disclosed here to mechanisms of eye disease, since ADAMTS9 is essential for eye development and is expressed along with MT1-MMP in the drainage apparatus, where it could have a role in intraocular pressure regulation (16, 85). In addition to fibronectin, previously identified as an ADAMTS9 substrate and other ECM and secreted proteins and proteoglycans, the present work also identified the cilium transition zone protein TMEM67, which like ADAMTS9, is mutated in ciliopathies as a potential substrate, warranting further investigation of TMEM67 processing in the context of ciliogenesis (86, 87, 88).

## Data Availability

The mass spectrometry proteomics data have been deposited to the ProteomeXchange Consortium *via* the PRIDE partner repository (89) with the dataset identifier PXD036612 and 10.6019/PXD036612. All identified proteins, peptides, and peptide spectral matches with scoring and spectral information can be found in supplemental Files S1–S4. Reviewer account details for the PRIDE dataset are: **Username**: reviewer_pxd036612@ebi.ac.uk; **Password**: u9qk5Bpm.

## Supplemental Data

This article contains supplemental data.

## Conflict of interest

The authors declare no competing interests.
