# Supplementary material for: Degradomic Identification of Membrane Type 1-Matrix Metalloproteinase as an ADAMTS9 and ADAMTS20 Substrate
Source: Mol Cell Proteomics. 2023 May 9;22(6):100566. doi: 10.1016/j.mcpro.2023.100566 (PMC10267602; doi:10.1016/j.mcpro.2023.100566)
Supplement: Supplemental information [file mmc9.docx]

**DATA SUPPLEMENT**

**Degradomic identification of membrane type 1-matrix metalloproteinase (MT1-MMP/MMP14) as an ADAMTS9 and ADAMTS20 substrate**

Sumeda Nandadasa*, Daniel Martin*, Gauravi Deshpande, Karyn L. Robert, M. Sharon Stack, Yoshifumi Itoh and Suneel S. Apte.

*Equal contribution.

**Content**:

This data supplement contains Table S1, Figures S1-S2 and descriptions of four movies (movies S1-S4).

Supplemental Files 1-4 provide detailed mass spectrometry data:

Supplemental File 1: Significant peptides identified in conditioned medium.

Supplemental File 2: All TAILS proteins, peptides and PSMs (Conditioned medium, dimethyl labeling).

Supplemental File 3: All preTAILS proteins, peptides and PSMs (Conditioned medium, dimethyl labeling).

Supplemental File 4: All preTAILS and TAILS proteins peptides and PSMs (Cell Lysate, iTRAQ labeling).

| TAILS experiment | Number of  peptides | N-terminally labeled peptides | K (Lys)- containing peptides^a^ | Labeling method | Labeled peptides^b^ | Labeling efficiency^c^ |
| --- | --- | --- | --- | --- | --- | --- |
| WT/D12 Medium TAILS | 1773 | 1587 (882 dimethyl,  225 glu, 480 acetyl) | 610 | Dimethyl | 593 (396 heavy/ 497 light) | 97% |
| WT/D12 Medium pre-TAILS | 5049 | 929 (731 dimethyl,  100 glu, 96 acetyl) | 2638 | Dimethyl | 2531 (988 heavy/ 1663 light) | 96% |
| WT/D12 Lysate  TAILS and pre-TAILS | 14032 | 2573 | 7244 | iTRAQ | 4821 | 67% |

**Table-S1: Labeling efficiency in TAILS experiments**

^a^K-peptides are peptides containing at least one lysine residue.

^b^Labeled peptides are designated as peptides carrying a dimethyl or iTRAQ tag on a lysine residue.

^c^Labeling efficiency is defined as the percentage of K-peptides carrying a dimethyl or iTRAQ tag on a lysine residue

**Figure S1.** Labeling efficiency for the dimethyl experiments was calculated based on the total number of PSMs with labeled lysines divided by the number of lysine-containing PSMs for each replicate in the TAILS and pre-TAILS analysis and is expressed as a percentage.

**
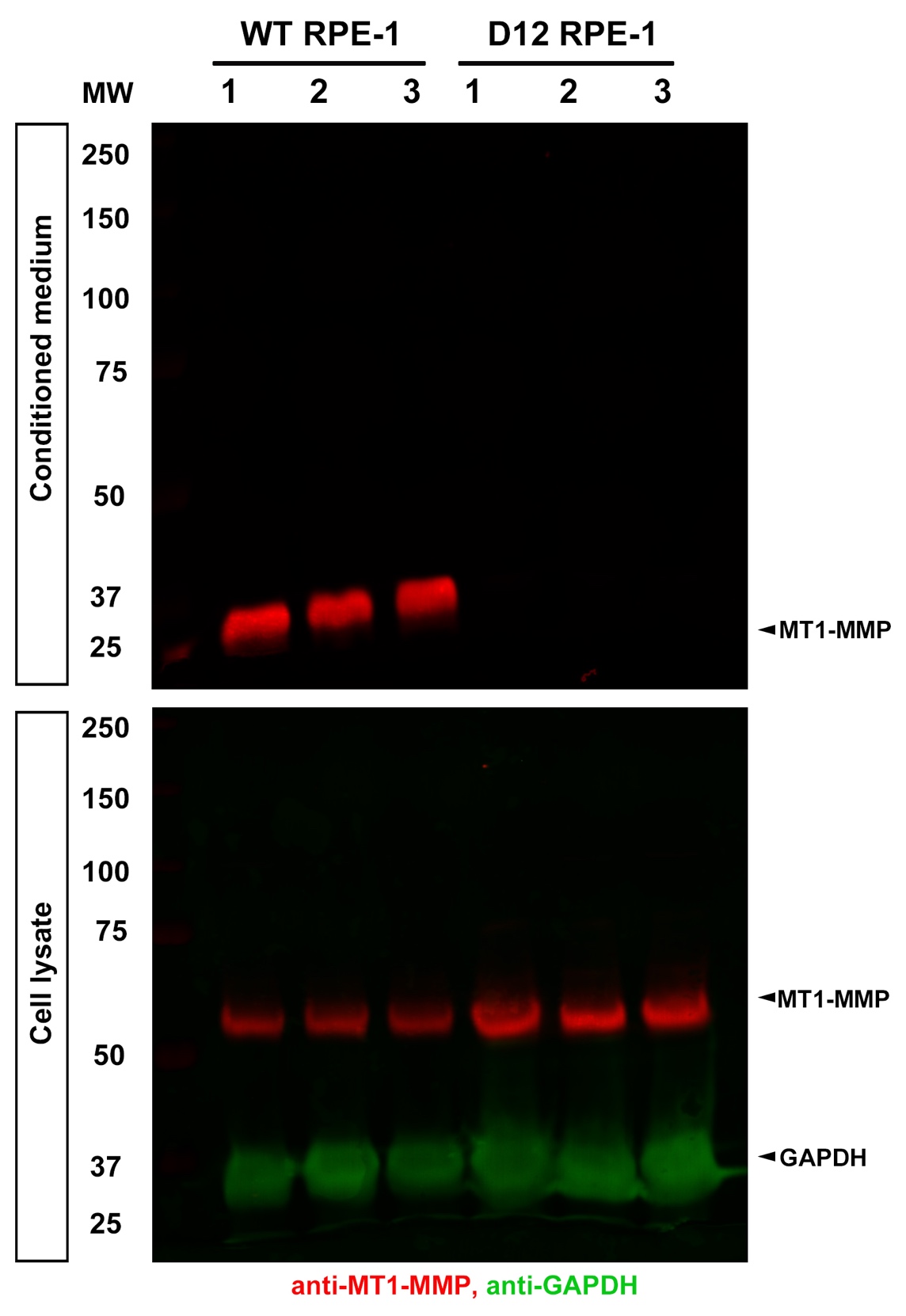
**

**Figure S2**. Wild type RPE-1 cells shed MT1-MMP catalytic domain to the conditioned medium while D12 cells fail to do so. Western blot of wild type and D12 cell conditioned medium (top) and cell lysate (bottom) co-stained for MT1-MMP (red) and GAPDH (green).


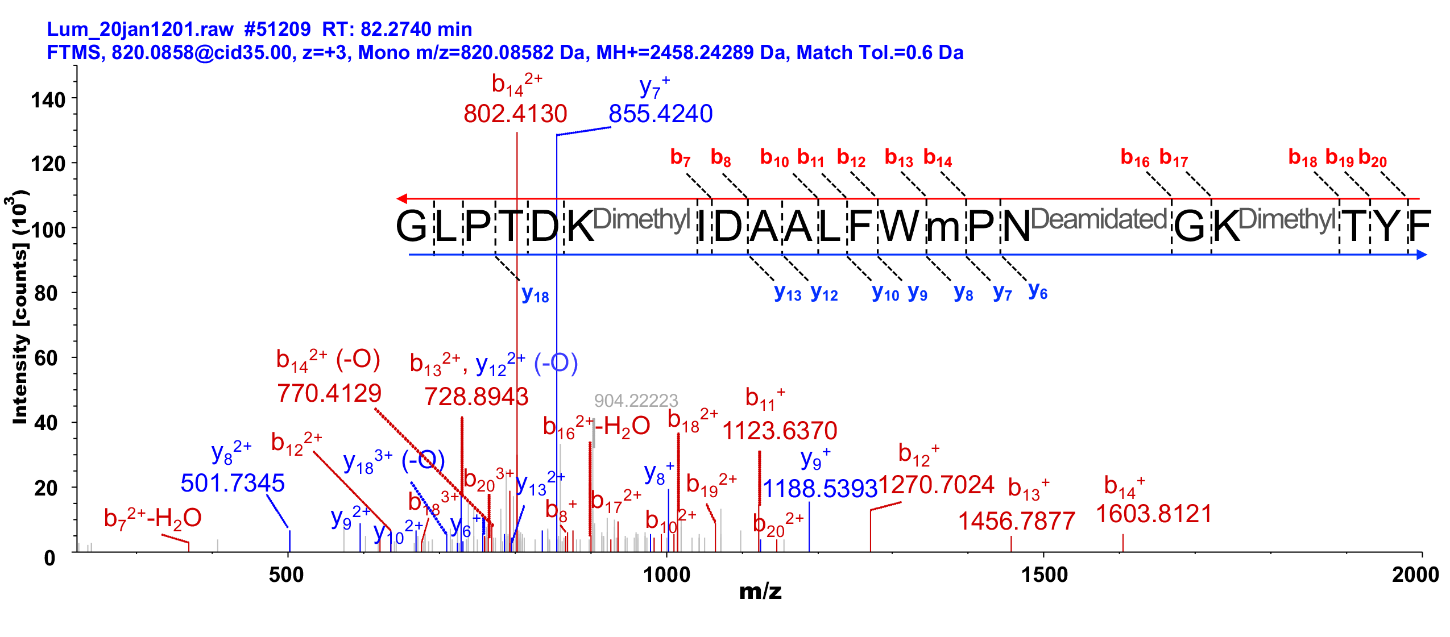


**Figure S3.** MS2 profile of the MT1-MMP peptide GLPTDKIDAALFWMPNGKTYF with an oxidized methionine that was also only found in wild-type digest.


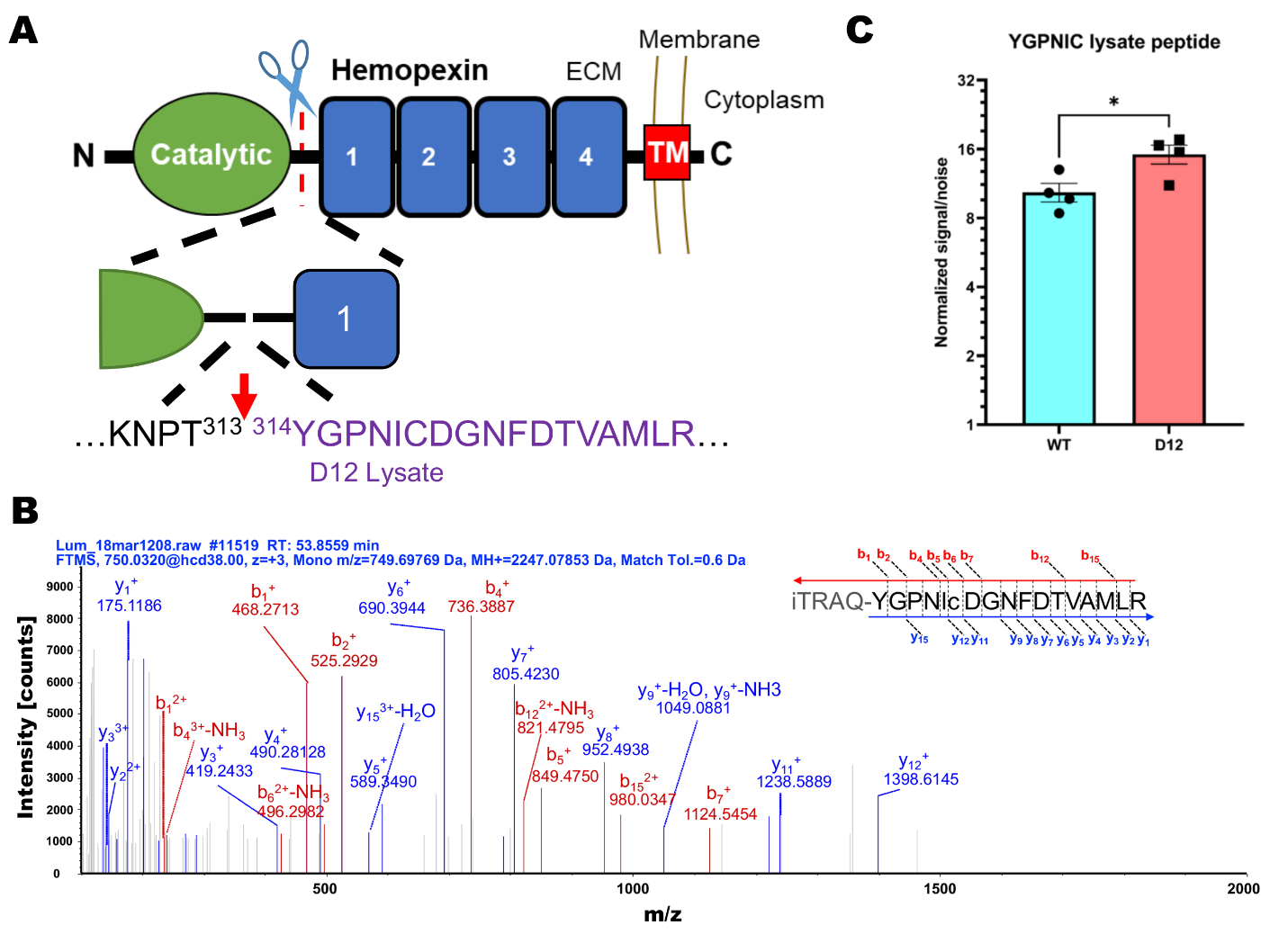


**Figure S4**. D12 cells have significantly higher MT1-MMP proteolysis at a distinct site in the hinge.

(A) Cartoon of furin-processed MT1-MMP showing the cleavage site. (B) MS2 spectrum of the peptide with an N-terminal iTRAQ label, whose sequence suggests the cleavage site. (C) Quantitation of the peptides in B showing higher levels in D12 cells than in parental RPE-1 cells.

**Supplemental Movies**:

Movies S1-S2: Time-lapse videos taken over a 2 h duration showing combined IRM and DIC microscopy of wild-type RPE-1 cells (Movie S1) and ADAMTS9-mutant D12 cells (Movie S2) taken 30 min after cell seeding. Cell adhesions on IRM are seen as dark areas superimposed on the cell boundaries visualized by DIC.

Movies S3-S4: The time-lapse videos were taken over a 2 h duration and show combined IRM and DIC microscopy of ADAMTS9-mutant D12 cells taken 24 h after cell seeding. Cell adhesions on IRM are seen as dark areas superimposed on the cell boundaries visualized by DIC. Movie S3 and S4 show cells transfected with a control siRNA or MMP14 siRNA respectively.
